# Pulmonary dust foci as rat pneumoconiosis lesion induced by titanium dioxide nanoparticles in 13-week inhalation study

**DOI:** 10.1186/s12989-022-00498-3

**Published:** 2022-09-14

**Authors:** Shotaro Yamano, Yuko Goto, Tomoki Takeda, Shigeyuki Hirai, Yusuke Furukawa, Yoshinori Kikuchi, Tatsuya Kasai, Kyohei Misumi, Masaaki Suzuki, Kenji Takanobu, Hideki Senoh, Misae Saito, Hitomi Kondo, Yumi Umeda

**Affiliations:** Japan Bioassay Research Center, Japan Organization of Occupational Health and Safety, Hadano, Kanagawa 257-0015 Japan

**Keywords:** Titanium dioxide nanoparticles (TiO_2_ NPs), Rat pneumoconiosis, Pulmonary dust foci (PDF), F344 rat, Whole-body inhalation

## Abstract

**Background:**

Most toxicological studies on titanium dioxide (TiO_2_) particles to date have concentrated on carcinogenicity and acute toxicity, with few studies focusing of pneumoconiosis, which is a variety of airspace and interstitial lung diseases caused by particle-laden macrophages. The present study examined rat pulmonary lesions associated with pneumoconiosis after inhalation exposure to TiO_2_ nanoparticles (NPs).

**Methods:**

Male and female F344 rats were exposed to 6.3, 12.5, 25, or 50 mg/m^3^ anatase type TiO_2_ NPs for 6 h/day, 5 days/week for 13 weeks using a whole-body inhalation exposure system. After the last exposure the rats were euthanized and blood, bronchoalveolar lavage fluid, and all tissues including lungs and mediastinal lymph nodes were collected and subjected to biological and histopathological analyses.

**Results:**

Numerous milky white spots were present in the lungs after exposure to 25 and 50 mg/m^3^ TiO_2_ NPs. Histopathological analysis revealed that the spots were alveolar lesions, characterized predominantly by the agglomeration of particle-laden macrophages and the presence of reactive alveolar epithelial type 2 cell (AEC2) hyperplasia. We defined this characteristic lesion as pulmonary dust foci (PDF). The PDF is an inflammatory niche, with decreased vascular endothelial cells in the interstitium, and proliferating AEC2 transformed into alveolar epithelial progenitor cells. In the present study, the AEC2 in the PDF had acquired DNA damage. Based on PDF induction, the lowest observed adverse effect concentration for pulmonary disorders in male and female rats was 12.5 mg/m^3^ and 6.3 mg/m^3^, respectively. The no observed adverse effect concentration for male rats was 6.3 mg/m^3^. There was a sex difference in lung lesion development, with females showing more pronounced lesion parameters than males.

**Conclusions:**

Inhalation exposure to TiO_2_ NPs caused PDF, an air-space lesion which is an alveolar inflammatory niche containing particle-laden macrophages and proliferating AEC2. These PDFs histopathologically resemble some pneumoconiosis lesions (pulmonary siderosis and hard metal pneumoconiosis) in workers and lung disease in smokers, suggesting that PDFs caused by exposure to TiO_2_ NPs in rats are an early pneumoconiosis lesion and may be a common alveolar reaction in mammals.

**Graphical Abstract:**

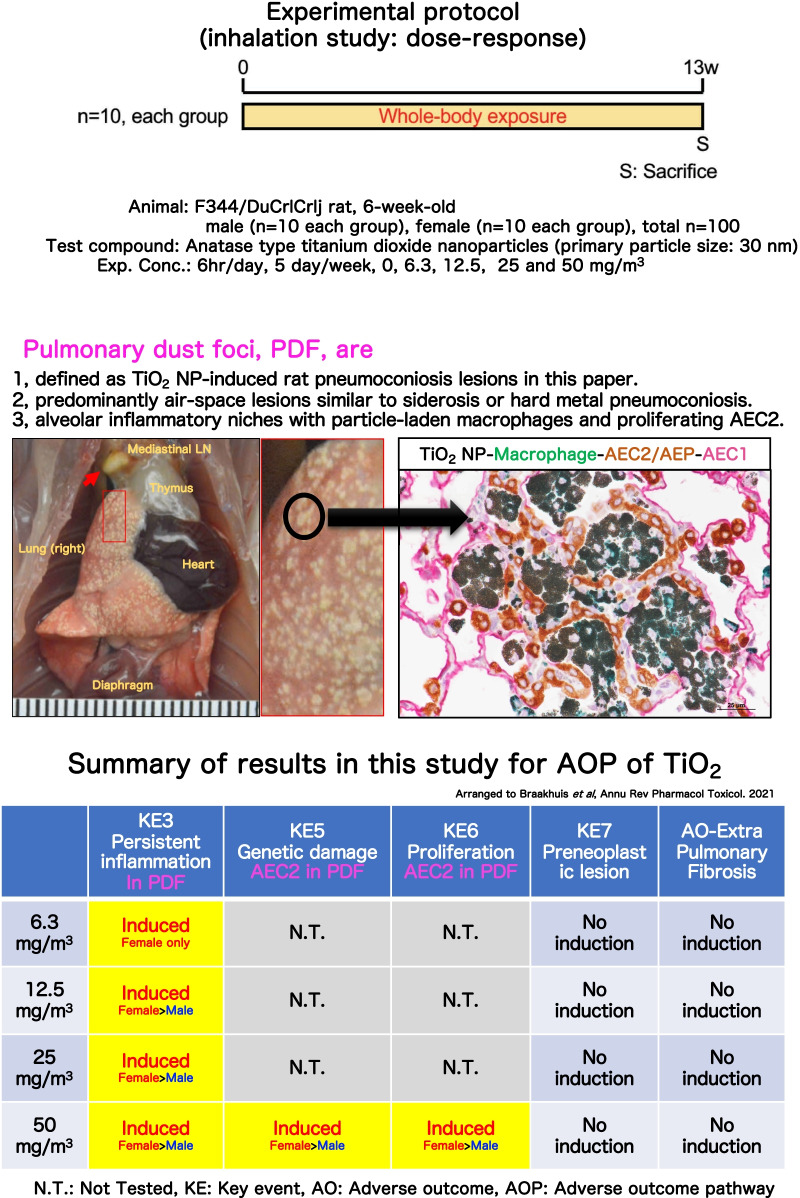

**Supplementary Information:**

The online version contains supplementary material available at 10.1186/s12989-022-00498-3.

## Background

Titanium dioxide nanoparticles (TiO_2_ NPs) have a variety of applications, from use in sunscreens, toners, and cosmetics to photodynamic therapy and treatment of waste water [1–4]. There are a variety of methods used to synthesize TiO_2_ NPs, resulting in the different particle properties that give TiO_2_ NPs their wide range of applications [1, 5, 6]. However, extensive use of TiO_2_ NPs without appropriate protections may lead to unexpected effects on human health, such as inhalation toxicity [7–10]. Indeed, there are a number of clinical case reports that workers exposed to TiO_2_ or titanium grindings suffered from various lung diseases [11–22]. Based on histopathological similarities, it is suggested that some of these cases include a type of pneumoconiosis, a typical occupational lung disease caused by inhalation of metal dust and fumes [23–26]. Pneumoconiosis is understood histopathologically as an airspace and interstitial lung disease caused by particle-laden macrophages; pneumoconiosis is chronic, progressive and still has no fundamental treatment [23–26]. Furthermore, the progression of pneumoconiosis is well known to increase the risk of lung cancer [27, 28]. Therefore, there is an urgent need to understand the toxicity mechanisms and pathogenesis of TiO_2_-associated pneumoconiosis to safe-guard the health of workers handling TiO_2_ NPs. However, most toxicological studies on TiO_2_ particles to date have focused on acute toxicity and carcinogenicity, and few studies have investigated the development of pneumoconiosis.

One subchronic inhalation toxicity study of TiO_2_ NPs (TiO_2_ obtained from DeGussa-Huls AG, designated P25 by the manufacturer, containing both anatase and rutile forms of TiO_2_, mean primary particle size 21 nm) conducted using rats, mice, and hamsters found that inhalation of 10 mg/m^3^ TiO_2_ NP for 13 weeks caused inflammatory responses in both rats and mice [29]. In addition, similarly to humans exposed to titanium grindings [22], rats developed progressive fibroproliferative lesions with interstitial particle accumulation, and alveolar septal fibrosis. These findings suggest that 13 weeks of inhalation exposure to rats is of sufficient duration to observe progressive lung lesions caused by TiO_2_ NPs and is an appropriate experimental protocol to assess the early stages of pneumoconiosis.

P25 contains mixture of two types of TiO_2_ crystal structures, anatase and rutile [30]. Therefore, the toxicities due to anatase TiO_2_ and rutile TiO_2_ were not distinguished from one another in the study by Bermudez et al. [29]. Furthermore, it has been reported that not only the crystal structure but also particle composition and surface characteristics such as surface passivation are important parameters for the pulmonary toxicity of TiO_2_ [31, 32].

In the present study, male and female rats were exposed to unmodified anatase TiO_2_ NPs for 13 weeks by systemic inhalation to investigate dose–response pathological changes associated with anatase TiO_2_ NPs, and to determine if exposure to anatase TiO_2_ NPs causes pneumoconiosis. We found that exposure to unmodified anatase TiO_2_ NPs did cause pneumoconiosis and were able to define the histopathological and cell biological basis of the development of pulmonary lesions associated with pneumoconiosis.

## Results

### Stability of aerosol generation and mass concentration and particle size distribution of TiO_2_ NPs in the inhalation chamber

The mass concentrations of TiO_2_ NP aerosol in the inhalation chambers are shown in Additional file 1: Fig. S1. The TiO_2_ NP concentrations were at the target concentrations over the 13-week exposure period: 6.37 ± 0.29 mg/m^3^ for the 6.3 mg/m^3^ group, 12.69 ± 0.87 mg/m^3^ for the 12.5 mg/m^3^ group, 25.04 ± 1.56 mg/m^3^ for the 25 mg/m^3^ group, and 49.89 ± 2.88 mg/m^3^ for the 50 mg/m^3^ group (Additional file 1: Fig. S1B).

The size distribution and morphology of the particles were measured at the first, sixth, and last week of exposure (Additional file 1: Fig. S1C). The size distribution were similar for all TiO_2_ NPs-exposed groups (Additional file 1: Fig. S1D). The mass median aerodynamic diameters (MMAD) and geometric standard deviations (σg) of the TiO_2_ NP aerosols were 0.9–1.0 μm and 2.0–2.1, respectively, and were similar for all TiO_2_ NP-exposed groups (Additional file 1: Fig. S1E-F). Morphological observations by scanning electron microscope (SEM) confirmed that the TiO_2_ NPs generated in the chamber were roughly spherical in shape and did not appear to be highly aggregated (Additional file 1: Fig. S1C). These data indicate that the size distribution and morphology of the TiO_2_ NP aerosols were consistent during the 13-week exposure period.

### Final body weights and organ weights

**Fig. 1:**
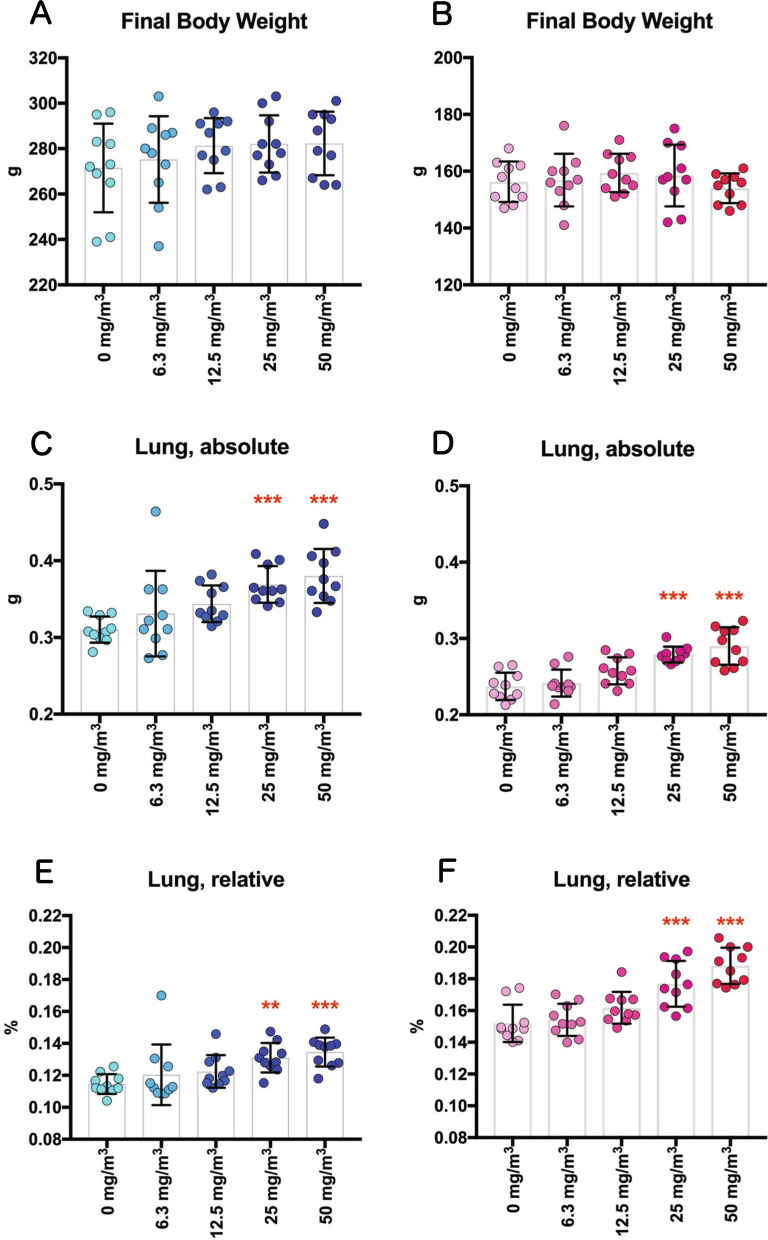
Final body weights and lung weights of F344 rats exposed to titanium dioxide nanoparticles (TiO_2_ NPs) by inhalation (6.3, 12.5, 25, or 50 mg/m^3^, 6 h/day, 5 days/week, 13 weeks). Final body weights of male (**A**) and female (**B**) rats (n = 10). Absolute left lung weights in male (**C**) and female (**D**) rats (n = 10). The relative lung weights in male (**E**) and female (**F**) rats were calculated as a percentage of body weight (n = 10). Dunn’s or Dunnett’s multiple comparison test: **p < 0.01, and ***p < 0.001

Neither exposure-related mortality nor respiratory clinical signs including dyspnea, irregular breathing or coughing were observed in any of the TiO_2_ NP-exposed rats throughout the study. There were no significant changes in final body weights (Fig. 1A, B, Additional file 13: Table S1). TiO_2_ NP concentration-dependent increases in lung weight were observed in both males and females (Fig. 1C–F, Additional file 13: Table S1). No statistically significant changes in the weight of organs other than the lungs were observed in any of the exposure groups (Additional file 14: Tables S2 and Additional file 15: S3).

### Blood hematology and biochemistry

Blood hematology and biochemistry data is shown in Additional file 16: Tables S4 and Additional file 15: S5. A significant increase in the percentage of eosinophils in the white blood cells (WBCs) was observed in males exposed to 12.5 mg/m^3^ and higher concentrations of TiO_2_ (Additional file 16: Table S4), and plasma lactate dehydrogenase (LDH) and aspartate aminotransferase (AST) activity and urea nitrogen levels were significantly increased in females in the 50 mg/m^3^ exposure group (Additional file 17: Table S5). However, while the changes were statistically significant, they were small and did not occur in both males and females, and therefore, these changes were judged to have low toxicological significance.

### Measurement of cytological and biochemical markers in the bronchoalveolar lavage fluid (BALF)

**Fig. 2:**
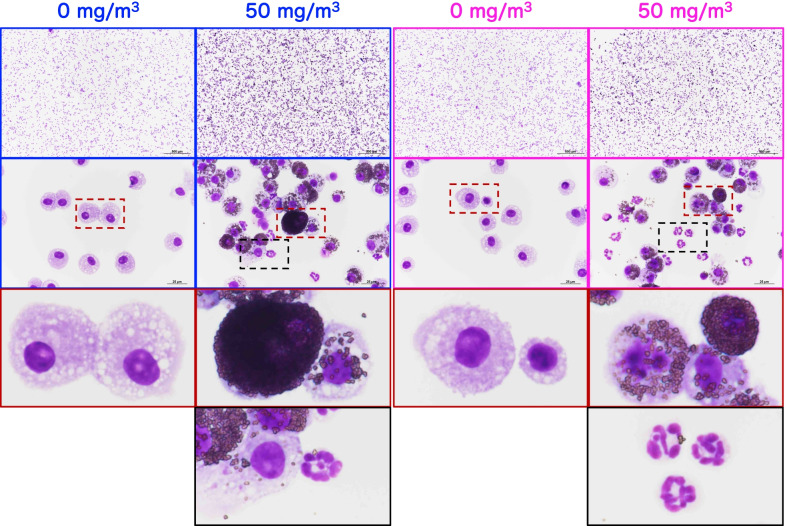
Representative images of the bronchoalveolar lavage fluid (BALF) cytospin cytology. The BALF samples obtained from males (blue) and females (pink) exposed to 0 mg/m^3^ or 50 mg/m^3^ TiO_2_ NPs were stained with May-Grunwald-Giemsa. The area enclosed by the red dotted square in the middle panel is enlarged and shown in the bottom panel. In the BALF of males and females exposed to 50 mg/m^3^, a marked increase in cell count was observed compared to their respective control groups (top panel of the figure). Particle-phagocytosing macrophages and neutrophils with segmental lobe nuclei (middle and lower panel of the figure) can be seen in the BALF of the TiO_2_ exposed rats

**Fig. 3:**
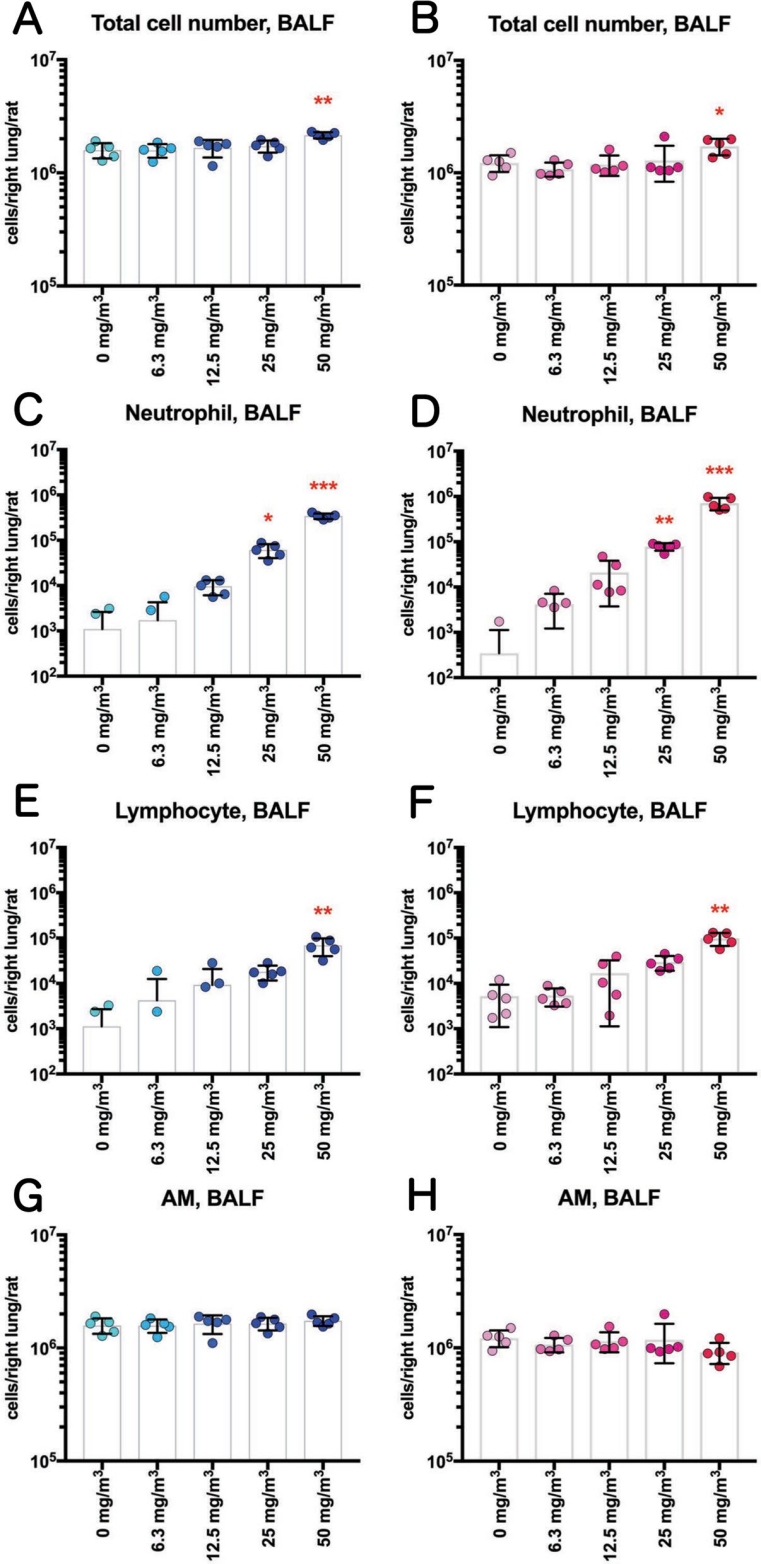
Effect of inhalation exposure to TiO_2_ NP on cell number in the BALF. The number of total cells (**A**, **B**), neutrophils (**C**, **D**), lymphocytes (**E**, **F**), and alveolar macrophages (AM) (**G**, **H**) were counted using an automated hematology analyzer, and are shown by sex (males: A, C, E and G; females: B, D, F and H) (n = 5). Statistical significance was analyzed using Dunn’s or Dunnett’s multiple comparison test: *p < 0.05, **p < 0.01, and ***p < 0.001

In the BALF of both male and female 50 mg/m^3^ exposure groups, neutrophils and enlarged macrophages phagocytosing TiO_2_ NPs were observed (Fig. 2). Cell population analysis found that the total cell number in the BALF of the male and female 50 mg/m^3^ exposure groups was significantly increased (Fig. 3A, B, Additional file 13: Table S1). Neutrophil numbers increased in a TiO_2_ NP concentration-dependent manner and were significantly increased in males exposed to 25 and 50 mg/m^3^ and in females exposed to 50 mg/m^3^ TiO_2_ (Fig. 3C, D, Additional file 13: Table S1). Lymphocyte numbers increased in a TiO_2_ NP concentration-dependent manner and were significantly increased in male and female 50 mg/m^3^exposure groups (Fig. 3E and F, Additional file 13: Table S1). In contrast, there was no increase in alveolar macrophage (AM) numbers in any of the exposure groups (Fig. 3G and H, Additional file 13: Table S1).

**Fig. 4:**
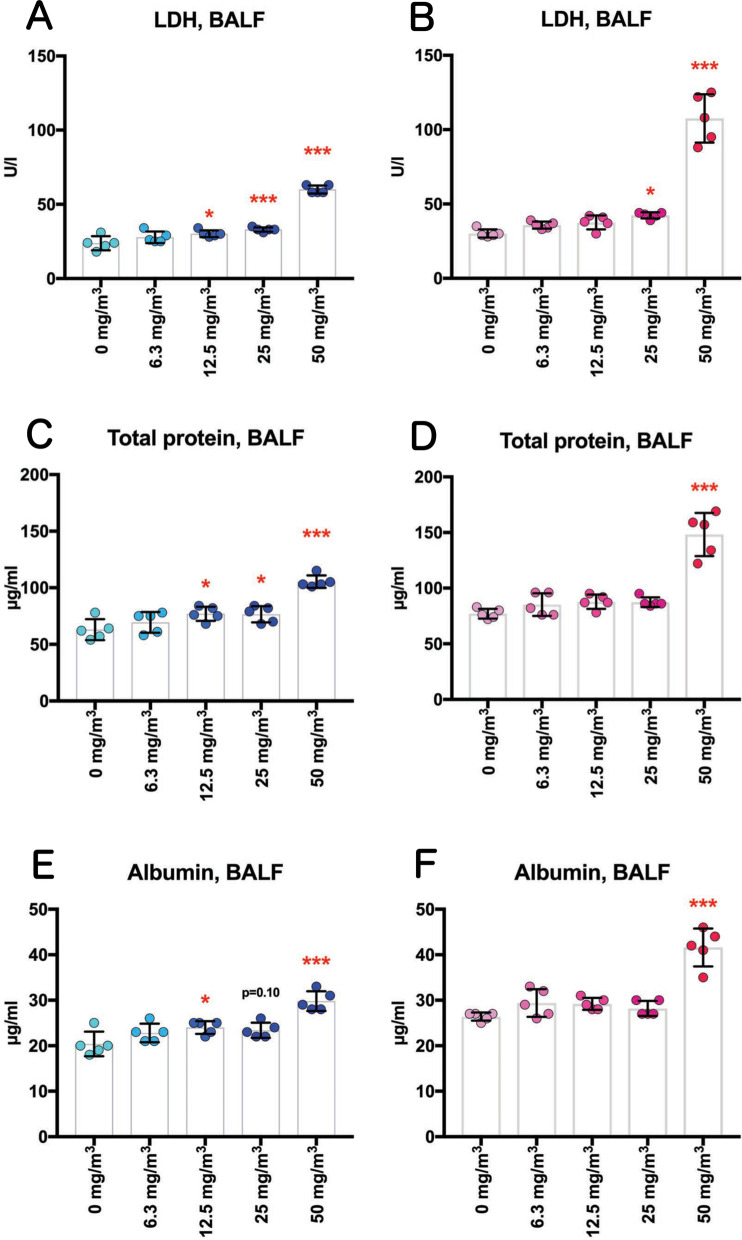
Dose-dependent induction of biochemical markers in the BALF obtained from the lungs of rats after inhalation of TiO_2_ NP for 13 weeks. Lactate dehydrogenase (LDH) activity (**A**, **B**), total protein concentration (**C**, **D**), and albumin concentration (**E**, **F**) in the BALF were measured using an automatic analyzer, and are shown by sex (males: A, C and E; females: B, D and F) (n = 5). Statistical significance was analyzed using Dunn’s or Dunnett’s multiple comparison test: *p < 0.05, and ***p < 0.001

TiO_2_ NP exposure increased LDH activity, total protein and albumin levels, but not alkaline phosphatase (ALP) or γ-glutamyl transpeptidase (γ-GTP) activities, in the BALF (Figs. 4 and S2, Additional file 13: Table S1). In males, these increases showed clear concentration dependence. In females, significant increases were only observed in the 50 mg/m^3^ group, however, in the 50 mg/m^3^ groups females showed more pronounced increases than males.

**Fig. 5:**
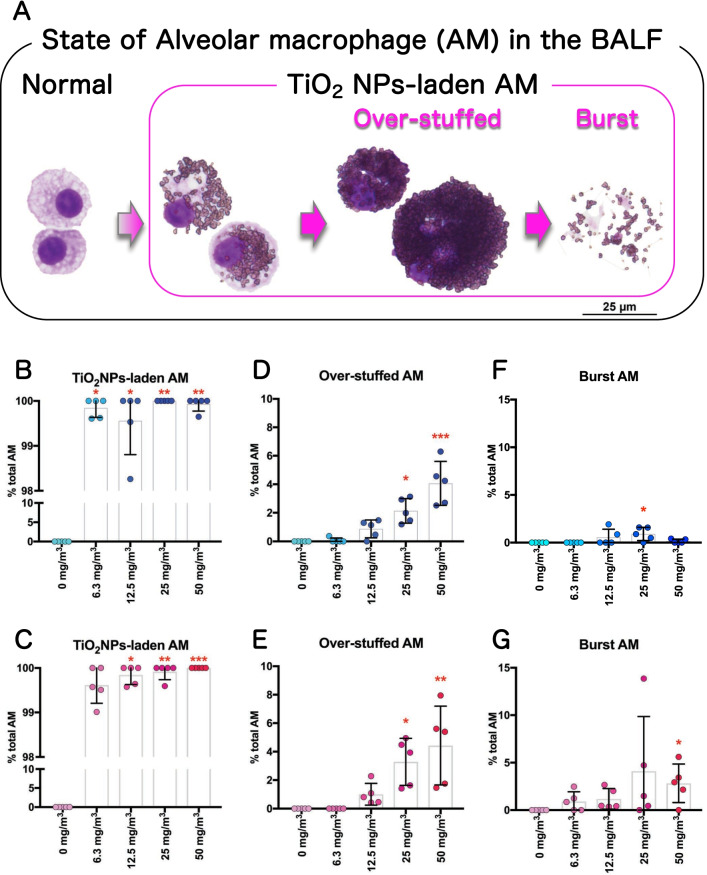
Additional analyses of alveolar macrophages (AMs) in BALF cytospin cytology. Various states of AMs phagocytosing TiO_2_ NPs were found by careful observation of BALF cytospin specimens (**A**). AMs that phagocytosed one or more TiO_2_ NPs were defined as TiO_2_ NP-laden AMs. AMs that phagocytosed TiO_2_ NPs until the nucleus was no longer visible were defined as Over-stuffed AMs. AMs that disintegrated into particles and cellular debris were defined as Burst AMs. The percentage of TiO_2_ NP-laden AMs (**B**, **C**), Over-stuffed AMs (**D**, **E**), and Burst AMs (**F**, **G**) were counted, and are shown by sex (males: B, D and F; females: C, E and G) (n = 5). Statistical significance was analyzed using Dunn’s or Dunnett’s multiple comparison test: **p < 0.01, and ***p < 0.001

In BALF cytospin specimens AMs were present in three TiO_2_ NP-phagocytic states (Fig. 5A). AMs that phagocytosed one or more TiO_2_ NPs were defined as TiO_2_ NP-laden AMs. TiO_2_ NP-laden AMs that phagocytosed TiO_2_ NPs until the nucleus was no longer visible were defined as Over-stuffed AMs, and AMs that disintegrated into particles and cellular debris were defined as Burst (Fig. 5A). On average, more than 99% of the AMs found in the BALF in all exposure groups of both sexes were TiO_2_ NP-laden (Fig. 5B, C, Additional file 13: Table S1). The percentage of Over-stuffed AMs increased in both sexes in an exposure concentration-dependent manner, with a statistically significant increase in the groups exposed to 25 and 50 mg/m^3^ (Fig. 5D, E, Additional file 13: Table S1). There was no concentration-dependent increase in the percentage of Burst AMs in the cytospin specimens from either sex (Fig. 5F, G, Additional file 13: Table S1).

### Lung burden and its correlation with lung weight and BALF markers

**Fig. 6:**
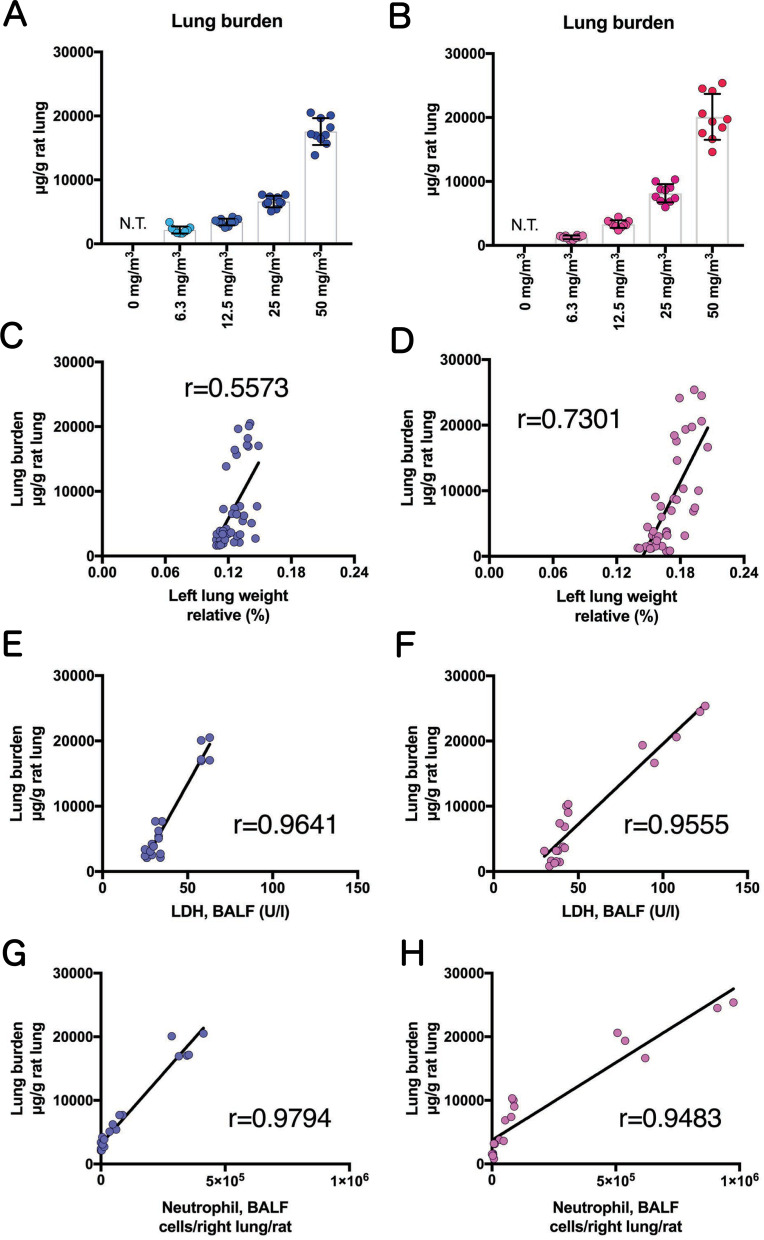
Lung burden and correlation between lung burden and relative lung weights and BALF markers. Lung burden of TiO_2_ NPs in male (**A**) and female (**B**) rats was measured by a Zeeman atomic absorption spectrometry (n = 10). Correlation between lung burden and relative lung weight (**C**, **D**), LDH activity in the BALF (**E**, **F**), and neutrophil number in the BALF (**G**, **H**) was analyzed using the Pearson’s correlation coefficients, and are shown by sex (male: C, E and G; female: D, F and H) (C and D: n = 10/each group, total n = 40, E–H: n = 5/each group, total n = 20). r: Pearson’s correlation coefficient. Abbreviation: N.T., not tested

OECD TG 413 recommends that lung burden should be measured when inhaled test particles are poorly soluble and are likely to be retained in the lungs [33]. Therefore, we investigated the correlation of lung burden with toxicological parameters. Lung burden measurements are shown in Fig. 6A, B and Additional file 13: Table S1. Inhalation of TiO_2_ NPs resulted in deposition of particles in the lungs in an exposure concentration-dependent manner, which tended to be higher in females than males in the 50 mg/m^3^ group (Fig. 6A, B). Relative lung weight, LDH activity in the BALF, and neutrophil count in the BALF were all positively correlated with lung burden in both sexes (Fig. 6C–H). The correlation plots of lung burden and LDH and lung burden and neutrophil count (Fig. 6E–H) appear to be divided into two clusters: data points from the 6.3, 12.5, and 25 mg/m^3^ exposed rats and data points from the 50 mg/m^3^ exposed rats.

### Macroscopic findings of lung and mediastinal lymph node

**Fig. 7:**
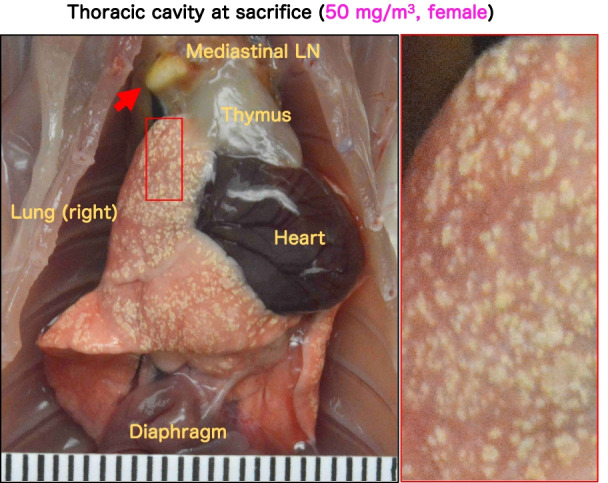
Representative macroscopic photographs of the thoracic cavity of female rats after 13-week of inhalation exposure to TiO_2_ NP (50 mg/m^3^, 6 h/day, 5 days/week). Scale bar: 1 mm. Abbreviation: LN, lymph node

Representative macroscopic images of the lungs and mediastinal lymph nodes are shown in Fig. 7 and Additional file 3: Fig. S3. In the lungs of the 25 and 50 mg/m^3^ exposed rats, a large number of milky white spots were observed in all lung lobes, mainly on the lung surfaces facing the ribs. The spots were generally approximately 300 nm in diameter, but some were partially fused and were about 1 mm in diameter (Fig. 7). The spots observed on the lung surface were fewer around the hilar region and more concentrated at the lung periphery (Additional file 3: Fig. S3B). The mediastinal lymph nodes also showed a similar color change (Fig. 7). However, no significant enlargement of mediastinal lymph nodes due to TiO_2_ NP exposure was observed. These gross changes in the lungs and mediastinal lymph nodes were observed only in the groups exposed to 25 and 50 mg/m^3^.

### Histopathological examination of the lungs and mediastinal lymph nodes

**Fig. 8:**
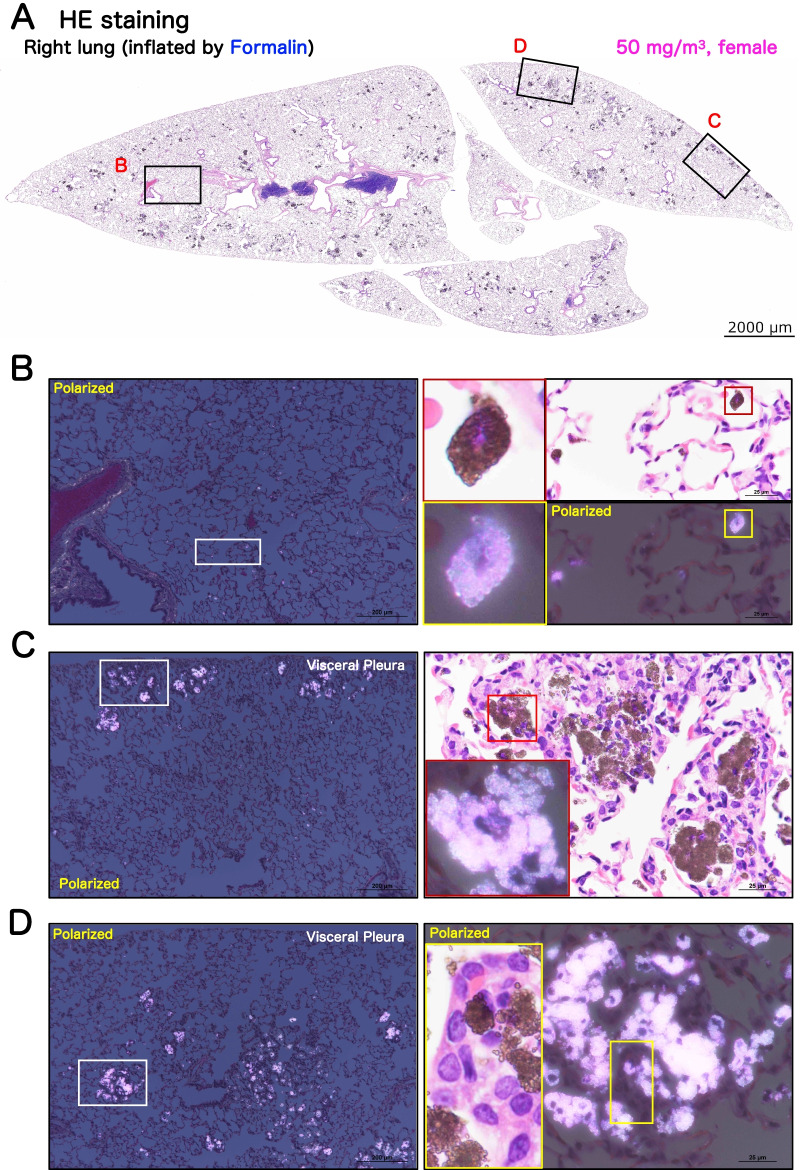
Representative microscopic photographs of female rat lungs after inhalation exposure to TiO_2_ NP (50 mg/m^3^). Representative loupe photograph (**A**) and magnified images of the area surrounding a lesion (**B**) and TiO_2_ NP-induced multifocal lesions (**C**, **D**) are shown. Formalin was injected into the right lung through the bronchus and the tissue was stained with hematoxylin and eosin (HE). TiO_2_ NPs phagocytosed by macrophages were observed throughout the alveolar region and were black in HE staining or birefringent using polarized light microscopy. A typical macrophage phagocytosing particles until the nucleus was not visible is shown on right side of panel **B**. Representative histological images of multifocal lesions in the area just below the pleura **C** and around the Bronchiolo-alveolar duct junction **D** are shown. Histopathologically, in each lesion particle-laden macrophage agglomeration was observed, and in many foci, alveolar epithelial proliferation and inflammatory cell infiltration was also observed (magnified right side of panels **C** and **D**).

Representative microscopic photographs and histopathological findings for the lungs and mediastinal lymph nodes are shown in Figs. 8, Additional file 4: Fig. S4, Additional file 5: Fig. S5, Additional file 6: Fig. S6 and Table 1. Deposition of particles in the alveolar air space (Fig. 8B), bronchus-associated lymphoid tissue (BALT) (Additional file 4: Fig. S4C), and mediastinal lymph node (Additional file 6: Fig. S6), which is commonly seen with inhalation exposure to particles, was observed in all groups exposed to TiO_2_ NPs (Table 1). Extrapulmonary ejection of TiO_2_ NPs via the mucociliary escalator was observed in all exposed groups (Additional file 4: Fig. S4B).

**Table 1:** Incidence and grade of the histopathological findings of the lung and mediastinal lymph nodes after inhalation exposure to TiO2 NP

Exposure concentration (mg/m^3^) No. of Animals Examined	Male					Female				
	0	6.3	12.5	25	50	0	6.3	12.5	25	50
	10	10	10	10	10	10	10	10	10	10
Histopathological findings										
Mediastinal lymph node										
Deposition of particles	0	3	6**	9***	10***	0	6**	7**	9***	10***
		< 1 >	< 1 >	< 1 >	< 1.5 >		< 1 >	< 1 >	< 1 >	< 1.5 >
Lung										
Deposition of particles: air space	0	10***	10***	10***	10***	0	10***	10***	10***	10***
		< 1 >	< 1 >	< 1 >	< 2 >		< 1 >	< 1 >	< 1 >	< 2 >
Deposition of particles: BALT	0	6**	9***	9***	10***	0	9***	9***	8***	10***
		< 1 >	< 1 >	< 1 >	< 1 >		< 1 >	< 1 >	< 1 >	< 2 >
Alveolar multifocal lesion										
Agglomeration of particle-laden macrophages: air space	0	0	7**	10***	10***	0	4*	9***	10***	10***
			< 1 >	< 1 >	< 2 >		< 1 >	< 1 >	< 1 >	< 2 >
Destruction of particle-laden macrophages	0	0	0	0	10***	0	0	0	0	9***
					< 1 >					< 1 >
Reactive AEC2 hyperplasia	0	0	1	5**	10***	0	2	4*	9***	10***
			< 1 >	< 1 >	< 2 >		< 1 >	< 1 >	< 1 >	< 2 >
Fibrosis, interstitial	0	0	0	0	0	0	0	0	0	0
Hyperplasia, bronchiolo-alveolar	0	0	0	0	0	0	0	0	0	0

Values indicate number of animals bearing lesions

The values in angle brackets indicate the average severity grade index of the lesion. The average severity grade is calculated with the following equation:

(grade X number of animals with grade)/number of affected animals

Grade: 1, slight; 2, moderate; 3, marked; 4, severe

BALT: Bronchus-associated lymphoid tissue

Significant difference: *, *p* < 0.05; **, *p* < 0.01; ***, *p* < 0.001 by Chi square test compared with the respective controls

The milky white spots on the lung surface observed by macroscopic observation were histopathologically identified as agglomerations of particle-laden macrophages accompanied by associated neutrophils and lymphocytes in the alveolar air spaces. We defined these lesions as multifocal lesions. Multifocal lesions were observed as black areas by HE staining (Figs. 8A and Additional file 4: S4A) and were birefringent under polarized light (Figs. 8B, C, D, Additional file 4: S4B, C, D). These multifocal lesions of the alveoli were located in the peripheral subpleural area (Fig. 8C), or in the alveolar region around the terminal bronchioles in the hilar region (Fig. 8D), and were induced in a concentration-dependent manner (Table 1). Particle-laden macrophages in the multifocal lesions phagocytosed TiO_2_ NPs to the extent that the nuclei were obscured, similar to the “Over-stuffed AM” observed in the BALF (Fig. 8C, D). Macrophages that had burst open, releasing their contents, were also observed in these multifocal lesions (Additional file 4: Fig. S4D). The presence of neutrophils and lymphocytes within the multifocal lesions suggest that these multifocal lesions are inflammatory niches.

**Fig. 9:**
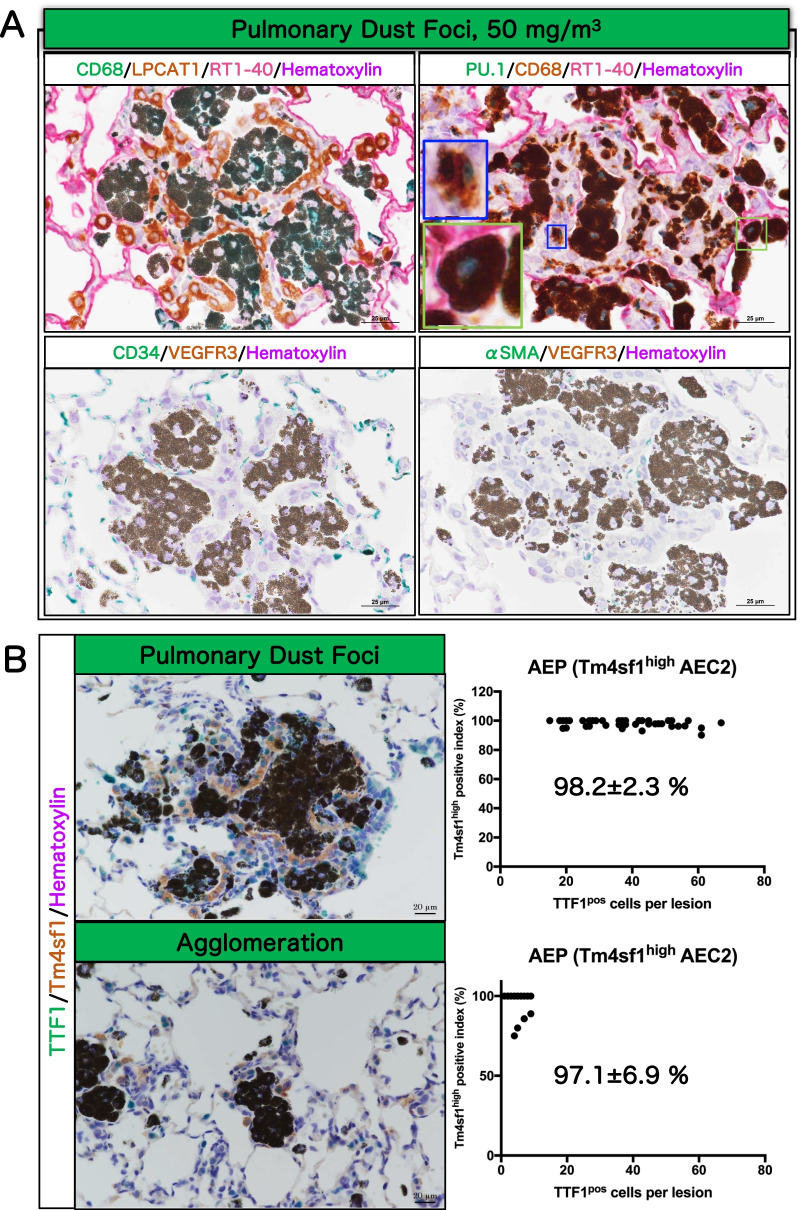
Immunohistochemical characteristics of pulmonary dust foci (PDF) and simple agglomeration lesions in rat lungs after inhalation exposure to TiO2 NP (50 mg/m^3^). The upper left of **A**: triple staining for macrophage marker CD68 (green in the cytoplasm), AEC2 marker LPCAT1 (brown in the cytoplasm) and AEC1 marker RT1-40 (red in the cell membrane). The upper right of **A**: triple staining for myeloid lineage marker PU.1 (green in the nucleus), CD68 (brown in the cytoplasm) and RT1-40 (red in the cell membrane). Blue frame: Particle-laden interstitial macrophage. Green frame: Over-stuffed AM in the alveolar air space. The lower left of A: double staining for vascular endothelial cell marker CD34 (green in the cell membrane) and lymphatic endothelial cell marker VEGFR3 (brown in the cell membrane). The lower right of A: double staining for myofibroblast marker αSMA (green in the cytoplasm) and VEGFR3 (brown in the cell membrane). Double staining for AEC2 marker TTF1 (green in the nucleus) and AEP marker Tm4sf1 (brown in the cytoplasm) in both PDF and simple agglomeration lesions (**B**). The percentage of TTF1/Tm4sf1 double positive AEPs in the total TTF1-positive cell population was measured for each of 50 randomly selected PDF lesions and 50 randomly selected simple agglomeration lesions, and shown as the Tm4sf1 positive index in AEC2 (mean ± S.D.)

Notably, proliferative changes in the alveolar epithelium were present in many of the multifocal lesions (Table 1; magnified view on the right in Fig. 8D). To confirm the cell types constituting these multifocal lesions, multiple staining with cell-specific markers was performed (Fig. 9, Additional file 7: Fig. S7, Additional file 8: Fig. S8 and Additional file 9: Fig. S9). The results showed that all the epithelial cells in the lesions were negative for the club cell marker club cell secretory protein (CCSP), the neuroendocrine cell marker calcitonin gene-related peptide (CGRP), the basal cell marker p63, and the bronchial epithelial lineage marker SRY-Box Transcription Factor 2 (Sox2) (Additional file 7: Fig. S7B). In contrast, the alveolar epithelium proliferating in multifocal lesions was positive for the alveolar epithelial type 2 cell (AEC2) markers lysophosphatidylcholine acyltransferase 1 (LPCAT1), Prosurfactant protein C (proSPC) and ATP-binding cassette transporter3 (ABCA3), indicating that AEC2 hyperplasia is co-localized with agglomeration of particle-laden macrophages in multifocal lesions (Fig. 9A and Additional file 8: Fig. S8). We defined this type of multifocal lesion containing AEC2 hyperplasia as "Pulmonary dust foci (PDF)".

Further examination revealed that PU.1 (nucleus) and CD68 (cytoplasm) double positive macrophages were present in the PDF (Fig. 9A). In addition to AMs (Green frame areas in Fig. 9A, upper right panel, show an "Over-stuffed AM" in the alveolar air space), interstitial macrophage infiltration was observed in the alveolar interstitium within the PDF (Fig. 9A, upper right panel), and particle-laden interstitial macrophages (Blue frame areas in Fig. 9A, upper right panel) were scattered in the alveolar interstitium.

The number of CD34-positive vascular endothelial cells was severely decreased in the alveolar interstitium within the PDF compared to the surrounding normal alveolar interstitium (Fig. 9A, lower left). Negligible vascular endothelial growth factor receptor 3 (VEGFR3)-positive lymphatic vessels (Fig. 9A lower panels) were observed in the PDF and α-smooth muscle actin (αSMA)-positive myofibroblasts (Fig. 9A, lower right) and collagen fibers (Additional file 10: Fig. S10) were not found, indicating decreased vascular endothelium in the PDF.

Transmembrane 4 superfamily member 1 (Tm4sf1)-positive AEC2 (Alveolar Epithelial Progenitor, AEP) appears during tissue regeneration of lung injury [34–36]. We determined the AEP index in the "Simple Agglomeration" lesions (multifocal lesions without AEC2 hyperplasia) and "PDF" lesions (multifocal lesions containing AEC2 hyperplasia) to ascertain whether AEP regeneration of lung injury was distinct in these two lesions. We found that the AEP positive index was the same in these lesions (Fig. 9B) and dramatically higher than in the surrounding normal alveolar region (Additional file 9: Fig. S9). These results suggest that development of multifocal lesions without AEC2 hyperplasia is an initial change leading to the development of PDF, and consequently, that the AEC2 to AEP transformation and epithelial proliferation in the PDF appear to be the result of a reaction to the particle-laden macrophages that constitute the multifocal lesion. Finally, the incidence and the number of PDF were examined. The incidence was significant in males exposed to 25 and 50 mg/m^3^ and in females exposed to 12.5, 25, and 50 mg/m^3^ TiO_2_ NPs (Table 2). PDF incidence and number showed an exposure concentration-dependent increase in both sexes. In addition, in the 50 mg/m^3^ exposure groups, there was a statistically significant increase in the number of PDF in females compared to males (Table 2), indicating that there is a sex difference in the development of PDF.

**Table 2:** Incidence and number of pulmonary dust foci

Concentration (mg/m^3^)	No. of rats	Pulmonary dust foci		
		Incidence (%)	Number (average ± SD)	
			No./slide	No./cm^2^ lung
*Male*				
0	10	0 (0)	0	0
6.3	10	0 (0)	0	0
12.5	10	1 (10)	0.10 ± 0.32	0.07 ± 0.21
25	10	5 (50) ^$$^	2.10 ± 2.60	1.12 ± 1.36
50	10	10 (100)^$$$^	76.40 ± 18.37***	47.85 ± 15.65***
*Female*				
0	10	0 (0)	0	0
6.3	10	2 (20)	0.20 ± 0.42	0.17 ± 0.36
12.5	10	4 (40)^$^	0.40 ± 0.52	0.33 ± 0.42
25	10	9 (90)^$$$^	7.80 ± 4.44	5.37 ± 3.23
50	10	10 (100)^0^	99.00 ± 25.43***,###	70.89 ± 21.03***,###

Significant difference: $, *p* < 0.05; $$, *p* < 0.01; $$$, *p* < 0.001 by Chi square test compared with the respective controls

^***^, *p* < 0.001 compared with the respective controls or ###, *p* < 0.001 compared with the male 50 mg/m^3^ group by two-way ANOVA with Tukey's multiple comparison test

### Cell proliferation activity of AEC2 in PDF

**Fig. 10:**
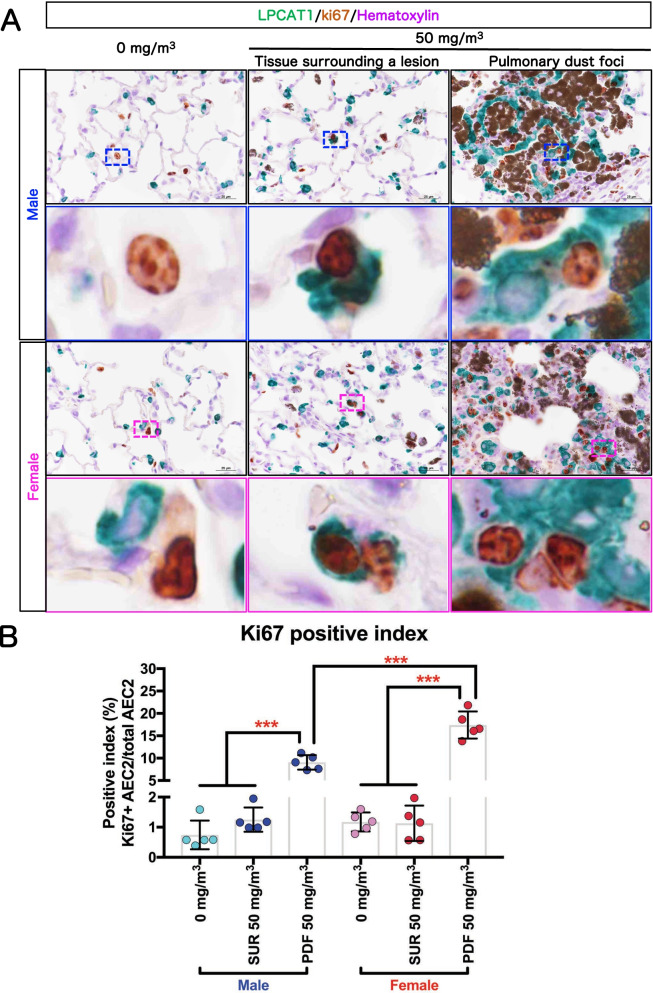
Cell proliferative activity of AEC2. Representative immunohistochemical staining images of Ki67, a cell proliferation marker, and LPCAT1, an AEC2 marker (**A**). The Ki67-positive index in AEC2 was calculated as the percentage of Ki67 and LPCAT1 double positive cells compared to the total LPCAT1-positive cell population (**B)** (n = 5). Statistical significance was analyzed using two-way ANOVA with Tukey’s multiple comparison test: ***p < 0.001 indicate significant differences. Abbreviations: PDF: pulmonary dust foci and SUR: tissue surrounding a lesion

As described in the previous section, in the PDF lesion AEC2 hyperplasia is co-localized with agglomeration of particle-laden macrophages. Since PDF is a major lesion caused by TiO_2_ NP inhalation, it is important to know the cell proliferative activity of the AEC2 cells within the PDF. We performed double staining for Ki67, a cell proliferation marker, and LPCAT1, an AEC2 marker, to evaluate the proliferation activity of AEC2 (Fig. 10). The results showed that the AEC2 Ki67-positive index in the PDF of both sexes in the 50 mg/m^3^ groups is significantly higher compared to both the alveolar area of rats in the clean air group (0 mg/m^3^) and in the tissue surrounding the lesions (SUR) (Fig. 10B). In addition, the Ki67-positive index within the PDF was significantly higher in females than in males. These results indicate that, in the PDF induced by TiO_2_ NPs, AEC2 has increased cell proliferative activity and that there is a sex difference in AEC2 proliferation.

### DNA damage in AEC2 in PDF

**Fig. 11:**
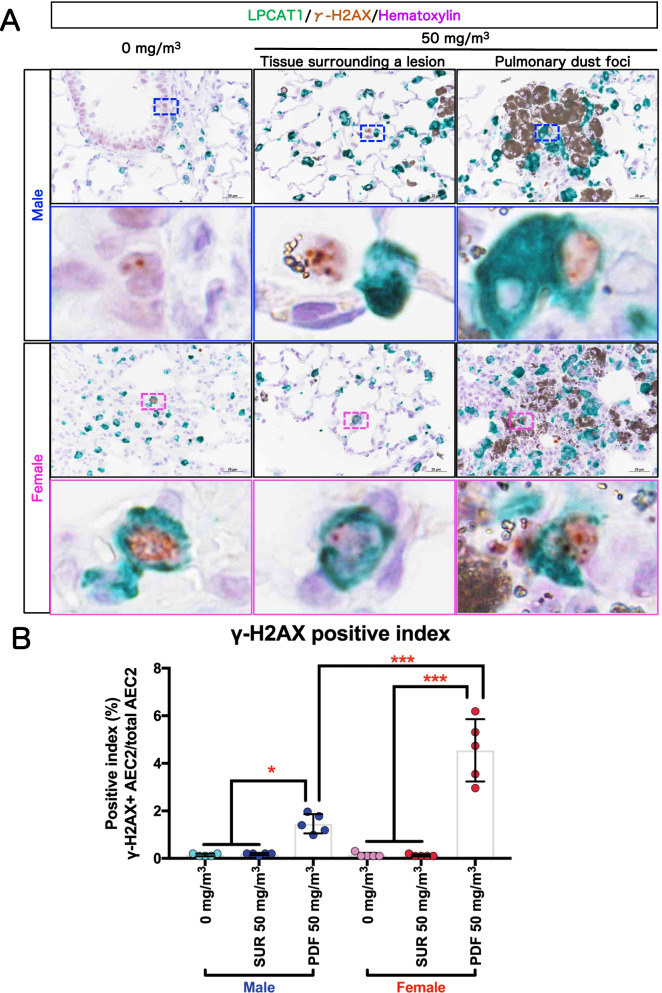
DNA damage in AEC2. Representative immunohistochemical staining images of the Ser-139 residue of the histone variant H2AX (γ-H2AX), a DNA double-strand break marker, and LPCAT1, an AEC2 marker (**A**). The γ-H2AX -positive index in AEC2 was calculated as the percentage of γ-H2AX and LPCAT1 double positive cells compared to the total LPCAT1-positive cell population (**B)** (n = 5). Statistical significance was analyzed using two-way ANOVA with Tukey’s multiple comparison test: *p < 0.05 and ***p < 0.001 indicate significant differences

It is postulated that TiO_2_ induced persistent inflammation leads to epithelial cell proliferation via injury to lung epithelial cells followed by tissue repair and that proliferation of epithelial cells with DNA damage leads to preneoplastic epithelial lesions [37]. Therefore, we assessed DNA damage in AEC2 by double staining for phosphorylation of the Ser-139 residue of the histone variant H2AX (γ-H2AX), a DNA double-strand break marker, and LPCAT1, an AEC2 marker (Fig. 11). The γ-H2AX-positive index of the AEC2 in the PDF was significantly increased in both sexes in the 50 mg/m^3^ groups compared to both the alveolar area of rats in the clean air group (0 mg/m^3^) and in the tissue surrounding the lesions (SUR) (Fig. 11B), and the γ-H2AX-positive index was significantly higher in females than in males. These results indicate that AEC2 in PDFs acquire DNA damage and that a higher proportion of cells are damaged in females. These results, taken together with the results presented above showing high AEC2 proliferation activity in the PDF, strongly suggest the presence of proliferating epithelial cells with DNA damage, which can lead to preneoplastic epithelial lesions [37].

### Histopathological findings in other organs

Histopathological findings in the nasal cavity, nasopharynx, heart, liver, kidney, pituitary gland, thyroid, testis, epididymis, prostate, oviduct, eye, Harderian gland, and bone marrow are shown in Additional file 18: Table S6. Inhalation exposure to TiO_2_ NPs caused toxic changes only in the nasal cavity and nasopharynx. In the nasal cavity and nasopharynx, goblet cell hyperplasia was observed in male rats exposed to 25 and 50 mg/m^3^ TiO_2_ NPs and in female rats exposed to 12.5, 25, and 50 mg/m^3^ TiO_2_ NPs. Eosinophilic changes in the olfactory and respiratory epithelium were induced in both sexes exposed to 12.5, 25, and 50 mg/m^3^ TiO_2_ NPs. Deposition of particles in the lymphoid tissue was observed in all exposed groups.

## Discussion

**Fig. 12:**
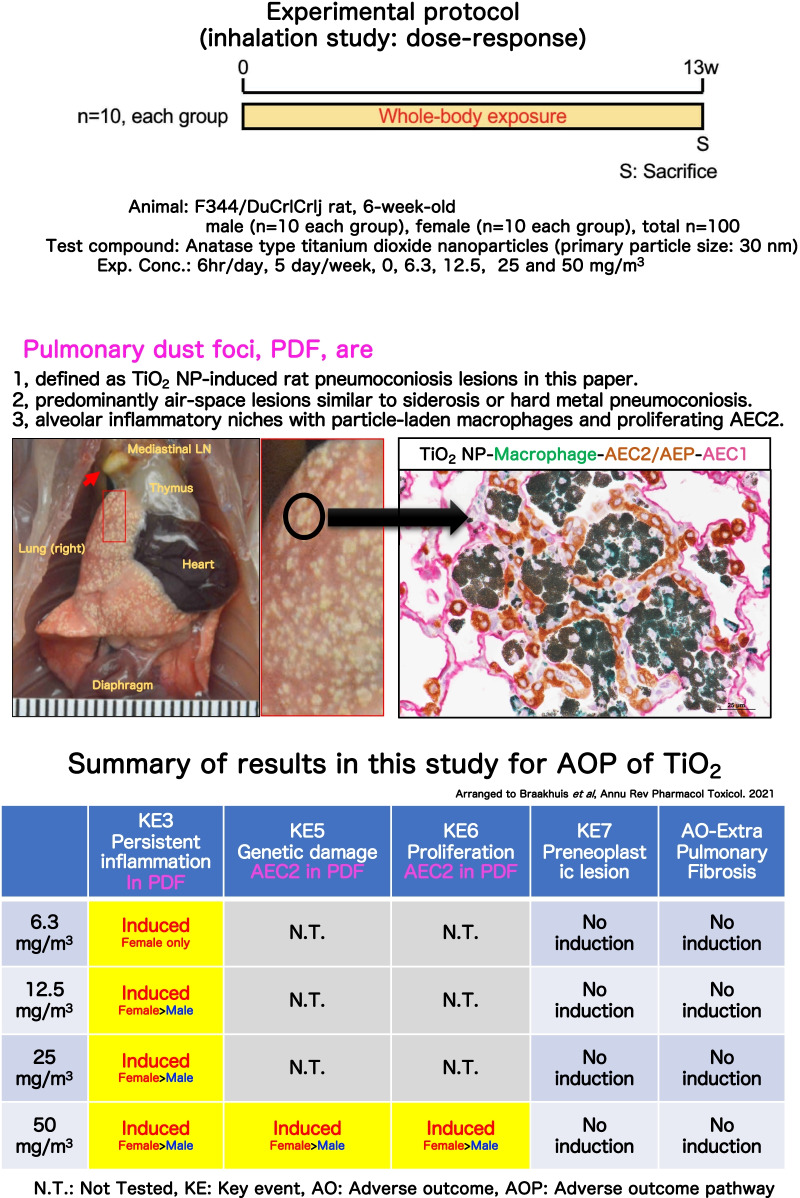
Graphical abstract in this study

We conducted a 13-week inhalation toxicity study of anatase TiO_2_ NPs according to OECD TG 413 guidelines. Male and female rats were exposed to 6.3, 12.5, 25 and 50 mg/m^3^ TiO_2_ NPs for 6 h per day, 5 days per week, for 13 weeks. Evaluation of all organs in the rats demonstrated that damage caused by inhalation exposure to TiO_2_ NPs was limited to the respiratory tract, especially the lung. The TiO_2_ NP exposed lungs showed multiple milky white spots on gross examination. Histopathological examination identified the main lesions as an agglomeration of particle-laden macrophages and AEC2 hyperplasia. We defined these lesion as pulmonary dust foci (PDF). As discussed below, PDF are likely to be early lesions associated with pneumoconiosis (Fig. 12).

The PDF has characteristics of an inflammatory niche with neutrophil and lymphocyte infiltration, decreased vascular endothelium, and particle-laden interstitial macrophage infiltration in the interstitium. The AEC2 in the PDF express Tm4sf1, indicating that they are Alveolar Epithelial Progenitor cells (AEP) and this type of cell contributes to alveolar regeneration [35, 36]. In agreement with this data, the AEP in the PDF had an increased Ki67-positive index indicating proliferative activity. PDF were observed in males exposed to 12.5 mg/m^3^ and higher concentrations of TiO_2_ NPs and in females exposed to 6.3 mg/m^3^ and higher concentration of TiO_2_ NPs. PDFs developed in both sexes in an exposure concentration-dependent manner. PDFs can be regarded as persistent inflammation and early development of PDFs suggest that they are key events in the pulmonary toxicity due to inhalation exposure to TiO_2_. Therefore, the lowest observed adverse effect concentration (LOAEC) for TiO_2_ NPs in this study was 12.5 mg/m^3^ for males and 6.3 mg/m^3^ for females. The no observed adverse effect concentration (NOAEC) was 6.3 mg/m^3^ for males. In females, since the lowest exposure concentration of TiO_2_ NPs caused the formation of PDF, the benchmark doses (BMDs) for the PDF were calculated using EPA's Benchmark Dose Software (BMDS 3.2). The result found that the benchmark dose lower confidence limit (BMDL) for female PDFs is around 1.5 mg/m^3^ (Additional file 19: Table S7). In agreement with this calculation, PDF-like lesions were also observed in a previously reported 13-week inhalation exposure study of TiO_2_ NPs (P25) using female rats, with a NOAEC of 2 mg/m^3^ based on pulmonary inflammation markers in the BALF [29].

The present study confirmed sex differences in TiO_2_ NP-induced lung toxicity at the highest dose of 50 mg/m^3^: increases in LDH activity, total protein, and albumin levels in the BALF and the incidence and number of Ki67- and γ-H2AX-positive indices in the PDF were higher in females than in males exposed to 50 mg/m^3^ TiO_2_ NP. Notably, the lung burden was higher in females than males in the 25 mg/m^3^ and 50 mg/m^3^ TiO_2_ NP exposure groups. To obtain further data, the MPPD particle deposition model was used to calculate the internal lung dose (Additional file 20: Table S8). Using this model, the TiO_2_ NP deposition per lung weight was lower in females than in males in all exposure groups. This indicates lower clearance of TiO_2_ NPs in females than in males in the 25 mg/m^3^ and 50 mg/m^3^ TiO_2_ NP exposure groups. However, the measured lung burden and the calculated internal lung dose were similar to or lower in females than in males in the 6.3 mg/m^3^ and 12.5 mg/m^3^ TiO_2_ NP exposure groups, and in these groups the incidence and number of PDF was higher in females than in males. This suggests that there is a sex difference in the onset and progression of TiO_2_ toxicity, with females being more susceptible to TiO_2_ toxicity than males. The fact that several toxicity indicators, including LDH activity, correlated positively with lung burden support this possibility.

To provide the data for risk management in the field, it is important to know the exposure limits extrapolated to humans for the adverse events obtained in this study. For this, the quantitative risk assessment (surface area criteria) by the National Institute for Occupational Safety and health (NIOSH) was used as a reference [38], and a computational extrapolation to human inhalation exposure concentrations was performed for PDF lesions in rats. Based on particle surface area per lung weight, benchmark dose confidence limits corresponding to a 1/1,000 excess PDF risk were calculated using BMDS 3.2, and converted to human exposure concentrations. Calculations based on the incidence of rat PDF lesions in male and female rats are shown in Additional file 21: Table S9: for a worker exposed to TiO_2_ NPs for 8 h/day, 5 days per week for 45-years, the exposure limits are 0.02 mg/m^3^ and 0.003 mg/m^3^ for males and females, respectively. While workers will not be exposed to TiO_2_ NPs for this length of time, reducing exposure to 1 h/day, 1 days/week for 2 years results in exposure limits of 2.65 and 0.4 mg/m^3^. Personal total exposure to 6.2 mg/m^3^ total TiO_2_ has been observed in packers, micronisers, and add-backs at TiO_2_ production plants [39]. As the grinding and filling processes in titanium dioxide manufacturing plants are generally observed to have the highest exposure levels and some workers are likely to have a short but high exposure or a long work history, appropriate safety controls may be necessary to address the pneumoconiosis risk to workers.

It is known from previous epidemiology and case reports of workers that pneumoconiosis can be caused by inhalation of various materials, including asbestos [40], silica [41], mixed dust [42], hard metals [43, 44], aluminum [45], beryllium [46], indium [47–49], and talcum [50]. In addition to the clinical findings of pneumoconiosis due to inhalation of the materials listed above, there have been many case reports of workers exposed to TiO_2_ particles including nanoparticles and titanium grindings [11–22]. The TiO_2_ associated lesions in workers' lungs ranged from alveolar lesions with agglomeration of particle-laden macrophages both in the air space and the alveolar interstitium, granulomas, alveolar fibrotic lesions such as nonspecific interstitial pneumonia (NSIP), bronchitis, and alveolar proteinosis. The variety of different lesions caused by exposure to TiO_2_ particles is likely due to the type of TiO_2_ particles the workers are exposed to. In addition, various confounding factors such as contamination of TiO_2_ particles with other minerals and smoking history, contribute to mixed reactions to inhaled TiO_2_ [23]. However, histopathological comparisons of rat lung lesions obtained in this study and pneumoconiosis of workers exposed to TiO_2_ demonstrate that several of these workers have alveolar lesions similar to the rat PDF described in the present study [18]. In addition, many workers' pneumoconiosis, such as arc-welders lung (also known as pulmonary siderosis) [51–54] and hard metal pneumoconiosis [43, 44, 55], have also been reported to have histopathological characteristics similar to the rat PDFs composed of both hypertrophic alveolar epithelial proliferation and alveolar filling macrophages. Furthermore, there are many clinical findings of alveolar lesions with PDF-like histopathology in idiopathic pulmonary hemosiderosis [56] and smoking-related lesions, such as smoking-related interstitial fibrosis (SRIF) [57, 58] and respiratory bronchiolitis interstitial lung disease/desquamative interstitial pneumonia (RBILD/DIP) [59–62]. Additionally, similar alveolar lesions occur in experimental animals such as rats and rabbits exposed by inhalation to different types of TiO_2_ [29, 63]. In summary, the PDF observed in this rat study is an early lesion of pneumoconiosis caused by exposure to TiO_2_, and is likely to be an alveolar reaction common to mammals. Further observation is necessary to determine whether PDF progresses to fibrotic interstitial lung disease over time.

In this study, we also examined whether the presumed key elements postulated to be caused by inhalation exposure to TiO_2_ occurred within the PDF (Fig. 12) [34]. For this analysis, we used lung samples from the male and female 50 mg/m^3^ exposure groups. We found inflammatory cells localized in the PDF, indicating that the PDF is an inflammatory niche where inflammatory cells infiltrate along with particle-laden macrophages, and is a "microenvironment" where persistent inflammation occurs. A significant increase in γ-H2AX and Ki67 positive indices in AEC2 in the PDF but not the surrounding area of the lesions provides clear evidence of genetic damage to lung epithelial tissue and AEC2 proliferation in the PDF lesions (Fig. 12). Our findings strongly support a mechanism whereby particle-laden macrophages become lodged in the alveolar airspace, which leads to persistent inflammation, persistent epithelial injury, and regenerative proliferation by AEC2.

The present study is the first report not only to define the histopathological and cell biological basis of TiO_2_ NPs-induced rat lung lesions caused by inhalation exposure to TiO_2_ NPs but also to clearly demonstrate increased DNA damage in AEC2: in the 50 mg/m^3^ exposure group, γ-H2AX expression was increased specifically in AEC2 in the PDF. Previous reports measuring TiO_2_ inhalation mediated DNA damage in the lung were negative [61, 62]. An important difference between these previous inhalation studies and the present study is that the previous studies assessed DNA damage in lung tissue and did not specifically assess DNA damage in AEC2. Studies using intratracheal instillation of TiO_2_ have also been reported, however, induction of DNA damage by TiO_2_ is not consistent among these studies [63–68]. As with the previous inhalation studies, five of these studies did not specifically examine DNA damage in AEC2 [63–66, 68]. While the study that did assess DNA damage in AEC2 did find TiO_2_ induced DNA damage [67], the extremely high amount of administered TiO_2_, 100 mg/kg, make this finding unreliable.

In the present study neither typical preneoplastic lesions nor pulmonary fibrotic lesions were observed in any of the male or female TiO_2_ NP exposure groups. As mentioned above, the PDF can be considered to be the initial lesion of TiO_2_ NP-induced pneumoconiosis in rats. Since the progression of pneumoconiosis in workers is well known to increase the risk of lung cancer [27, 28], it is important to investigate whether lung cancer in rats will develop as a complication of PDF development. To address this issue, we are currently conducting a 104-week systemic inhalation study using F344 rats.

## Conclusions

Inhalation exposure to TiO_2_ NPs for 13 weeks induced pulmonary lesions triggered by particle-laden macrophages in the alveoli of the F344 rat lung. We defined this specific lesion as pulmonary dust foci (PDF). The TiO_2_ NP-induced rat PDF is an inflammatory niche in the lung. Persistent inflammation causes tissue damage and induces AEC2 transformation to alveolar epithelial progenitor cells (AEP) which proliferate to repair inflammation mediated tissue damage. In the presence of inflammatory mediators AEP cells acquire DNA damage. Based on PDF induction, the LOAEC for pulmonary disorders in male and female rats in this study was 12.5 mg/m^3^ and 6.3 mg/m^3^, respectively. There was a sex difference in the lung lesions onset, with females showing more progressive lesion parameters than males. The similar histopathology to human pneumoconiosis makes it is highly likely that the PDF in the rat is an early lesion of rat pneumoconiosis. Further studies should focus on the progression of PDF over time for better understanding of TiO_2_-NP-inhalation-mediated pneumoconiosis and its carcinogenic potential. Importantly, different TiO_2_ particles will have different toxicities. For example, LDH activity in rat BALF after inhalation exposure to anatase TiO_2_ NPs (this study) was lower than that of ultrafine TiO_2_ particles (P25) reported in a previous study [29] (Additional file 22: Table S10), suggesting that inhalation of the anatase type TiO_2_ NPs used in this study is less harmful than inhalation of P25.

## Materials

Anatase type nano-titanium dioxide, TiO_2_ NP (aNTiO_2_) (Additional file 11: Fig. S11) was purchased from Tayca co. (primary particle size: 30 nm). TiO_2_ NP characteristics are summarized in Additional file 23: Table S11. A list of all primary antibodies used in these studies is shown in Additional file 23: Table S12. Other reagents used in the study were of the highest grade available commercially.

### Animals

Male and female F344 rats at 4 weeks old were purchased from Charles River Laboratories Japan, Inc. (Kanagawa, Japan). The rats were housed in an air-conditioned room under a 12 h light/12 h dark (8:00–20:00, light cycle) photoperiod, and fed a general diet (CR-LPF, Oriental Yeast Co. Ltd., Tokyo, Japan) and tap water ad libitum. After a 1 week quarantine and acclimation period, they were exposed to TiO_2_ NP. All animal experiments were approved by the Animal Experiment Committee of the Japan Bioassay Research Center.

### Generation of TiO_2_ NP aerosol

The generation of TiO_2_ NP aerosol into the inhalation chamber was performed using our established method (cyclone sieve method) [72, 73] with some modifications. Briefly, TiO_2_ NP was fed into a dust feeder (DF-3, Shibata Scientific Technology, Ltd., Soka, Japan) to generate TiO_2_ NP aerosol, and the aerosol was introduced into a particle generator (custom-made by Seishin Enterprise Co., Ltd., Saitama, Japan) to separate the aerosol and feed it into the inhalation chamber. The concentration of the TiO_2_ NP aerosol in the chamber was measured and monitored by an optical particle controller (OPC; OPC-AP-600, Shibata Scientific Technology), and the operation of the dust feeder was adjusted by feedback control based on upper and lower limit signals to maintain a steady state.

The mass concentration of TiO_2_ NP aerosol in the chamber was measured every two weeks during the exposure period. Aerosols collected on a fluoropolymer binder glass fiber filter (T60A20, φ55 mm, Tokyo Dylec, Corp., Tokyo, Japan) were weighed for each target concentration at 1, 3, and 5 h after the start of exposure. Using the mass per particle (K-value) calculated using the measured mass results (mg/m^3^) and the particle concentration data (particles/m^3^) obtained from the OPC, the particle concentration for each group during the exposure period was converted into mass concentration. The particle size distribution and morphology of the TiO_2_ NPs were measured at the 1st, 6th, and 13th weeks of exposure. The particle size distribution was measured using a micro-orifice uniform deposit cascade impactor (MOUDI-II, MSP Corp., Shoreview, MN). The MMAD and σg were calculated by cumulative frequency distribution graphs with logarithmic probability (Additional file 1: Fig. S1E). The TiO_2_ NPs in the inhalation chamber were collected on a 0.2 μm polycarbonate filter (φ47 mm, Whatman plc, Little Chalfont, UK), and observed using SEM (SU8000, Hitachi High-Tech, Tokyo, Japan) (Additional file 1: Fig. S1C).

### 13-week inhalation study

This experiment was conducted with reference to the OECD Guideline for Testing of Chemicals (TG 413) [74]. Based on the results of a dose-finding study conducted previously and OECD TG 413, target concentrations for TiO_2_ NP aerosols were set at 6.3, 12.5, 25, and 50 mg/m^3^, and the exposure schedule was 6 h per day, 5 days per week, for 13 weeks (Additional file 12: Fig. S12). One hundred rats (10 males and 10 females in each group) were transferred to individual stainless steel cages and exposed to TiO_2_ NP for 6 h with access to food and water. Animals were autopsied on two separate days beginning the day after the final exposure date (approximately 50 animals/day). All animals were fasted from the day before the autopsy date. Rats were exsanguinated, and the following sampling was performed: BALF was collected from 5 males and 5 females from each group sacrificed on the first day and blood was collected from 5 males and 5 females from each group sacrificed the next day, as described below. For histopathological analysis, all tissues were collected from all of the rats in each group, and fixed in 10% neutral phosphate buffered formalin solution.

### BALF collection and analysis

The left bronchus was tied with a thread, and the right lung was lavaged: 4–5 ml of saline was injected into the lung through the trachea, in and out twice, and collected as BALF. The total cell numbers in the BALF were counted using an automatic cell analyzer (ADVIA120, Siemens Healthcare Diagnostics Inc. Tarrytown, NY). Cell populations were prepared on glass slides using Cytospin 4 (Thermo Fisher Scientific, Inc., Waltham, MA). After May-Grunwald-Giemsa staining, differential white blood cell count was made by visual observation. BALF cytospin specimens were carefully examined under a microscope to classify the status of AMs phagocytosing TiO_2_ NPs. All AMs were divided into TiO_2_ NPs-laden AMs and normal AMs. The TiO_2_ NPs-laden AMs were then classified as Over-stuffed AMs, which had phagocytosed TiO_2_ NPs until the nucleus was no longer visible and Burst AMs, which were disintegrated into particles and cellular debris, and the number of each type of AM was counted.

The BALF was centrifuged at 1,960 rpm (800 × *g*) for 10 min at 4 °C, and the activity of LDH, ALP and γ-GTP, and the level of total protein and albumin in the supernatant was measured using an automatic analyzer (Hitachi 7080, Hitachi, High-Tech Corp., Tokyo, Japan).

### Titanium burden analysis

To determine the lung burden of Ti in TiO_2_ NP-exposed rats, approximately 0.1 g of lung tissue was collected and weighed. The lung tissue was put into a glass vessel, treated with 3 mL of distilled water, 3 mL of sulfuric acid, and 1 mL of nitric acid at 270 °C for 1 h. Samples were then diluted to 30–50 mL with 3% sulfuric acid. The samples were further diluted 2 to 50 fold to keep the concentration within the calibration curve, and TiO_2_ concentration in the samples was determined by Zeeman atomic absorption spectrometry (Z-5010; Hitachi High-Tech Corporation, Tokyo, Japan) with a Hitachi High-Tech lamp for Ti (part#207–2012 Serial 0,490,158,100). Absorbance of the digested samples was detected at 364.3 nm. Quantification was performed using a seven point calibration curve prepared by diluting appropriate volumes of a 1000 mg/L stock solution (Kanto Chemical Co., Inc., Tokyo, Japan) to 0.025, 0.05, 0.1, 0.15, 0.2, 0.3, and 0.4 µg/ml. TiO_2_ concentrations were calculated from the corresponding molecular weight ratio of TiO_2_ to Ti. The values obtained were calculated as the amount of Ti per gram. The correlation between the lung burden and several toxicological markers was calculated using the Pearson correlation coefficient (Pearson’s r) using GraphPad Prism 5 (GraphPad Software, San Diego, CA).

### Hematological and blood chemistry tests

For hematological examination, blood samples collected at the time of each autopsy were analyzed with an automated hematology analyzer (ADVIA120, Siemens Healthcare Diagnostics Inc. Tarrytown, NY). For biochemical tests, the blood was centrifuged at 3,000 rpm (2,110 × *g*) for 20 min, and the supernatant was analyzed with an automated analyzer (Hitachi 7080, Hitachi, Ltd., Tokyo, Japan).

### Histopathological analysis

Serial tissue sections were cut from paraffin-embedded lung specimens, and the first Sect. (2-μm thick) was stained with H&E for histological examination and the remaining sections were used for immunohistochemical analysis. The histopathological findings in this study were determined by certified pathologists from the Japanese Society of Toxicologic Pathology, based on terms adopted by International Harmonization of Nomenclature and Diagnostic Criteria for Lesions in Rats and Mice (INHAND)[75]. Pathological diagnosis was performed blindly by three pathologists and summarized. Each non-neoplastic lesion was evaluated for its severity and scored on a scale of “slight” to “severe” with reference to the criteria by Shackelford et al. [76].

### Masson’s Trichrome staining

Details of this procedure have been described previously [34]. Briefly, the slides were deparaffinized, washed with water, and then reacted with an equal volume of a mixture of 10% potassium dichromate and 10% trichloroacetic acid for 60 min at room temperature. The specimens were then washed with water and stained with Weigelt’s iron hematoxylin solution (C.I.75290, Merck-Millipore) for 10 min at room temperature. Specimens were then successively stained with 0.8% orange G solution (C.I.16230, Merck-Millipore) for 10 min at room temperature, Ponceau (C.I.14700, FUJIFILM-Wako Pure Chemical Corp., Osaka, Japan) acid fuchsin (C.I.42685, Merck-Millipore) azofloxine (C.I.18050, Chroma Germany GmbH, Augsburg, Germany) mixture for 40 min at room temperature, 2.5% phosphotungstic acid for 10 min at room temperature, and blue aniline solution (C.I.42755, Chroma Germany GmbH) under a microscope until color developed. Between each staining solution the slides were washed lightly with 1% acetic acid in water. Then, dehydration, permeabilization, and sealing were performed.

### Elastica Van Gieson staining

Briefly, the slides were deparaffinized, washed with water, reacted with Maeda Modified Resorcinol-Fuchsin Staining Solution (Mutoh Chemical, Part No. 40321, Japan) for 30 min at room temperature, and rinsed with 100% ethanol to remove excess stain. The slides were then washed with running water, stained with Weigelt's iron hematoxylin solution (C.I.75290, Merck-Millipore, US) for 10 min at room temperature, and washed with running water for 10 min. The slides were then reacted with 1% Sirius red solution (Mutoh Chemical, Part No. 33061, Japan) for 3–5 min at room temperature, washed with water, dehydrated with 90%-100% ethanol, permeabilized, and sealed.

### Immunohistological multiple staining analyses

Details of the multiple staining method have been described previously [77]. Briefly, lung tissue sections were deparaffinized with xylene, hydrated through a graded ethanol series, and incubated with 0.3% hydrogen peroxide for 10 min to block endogenous peroxidase activity. Slides were then incubated with 10% normal serum at room temperature (RT) for 10 min to block background staining, and then incubated for 2 h at RT with the first primary antibody. After washing with PBS, the slides were incubated with histofine simple stain rat MAX-PO (MULTI) (414,191, Nichirei, Tokyo, Japan) for 30 min at RT. After washing with PBS, slides were incubated with DAB EqV Peroxidase Substrate Kit, ImmPACT (SK-4103, Vector laboratories) for 2–5 min at RT. Importantly, after washing with dH_2_O after color detection, the sections were treated with citrate buffer at 98 °C for 30 min before incubation with the next primary antibody to denature the antibodies already bound to the section. This procedure was repeated for the second and then the third primary antibody. HighDef red IHC chromogen (ADI-950–142, Enzo Life Sciences, Inc., Farmingdale, NY) was used for the second coloration and Histogreen chromogen (AYS-E109, Cosmo Bio, Tokyo, Japan) for the third coloration. Coloration was followed by hematoxylin staining for 30–45 s. The slides were then processed for light microscopy. The sections were observed under an optical microscope ECLIPSE Ni (Nikon Corp., Tokyo, Japan) or BZ-X810 (Keyence, Osaka, Japan).

To perform various morphometric measurements on PDFs, only the 50 mg/m^3^ group of both sexes, which could ensure a sufficient number of PDF occurrences to be analyzed, were used in this study.

For measurement of Ki67 and γ-H2AX positive indices, the male and female 0 mg/m^3^ groups (n = 5) and the 50 mg/m^3^ groups (n = 5) were used for analysis. For the 50 mg/m^3^ exposure groups, positive indexes were counted separately for pulmonary dust foci (PDF) and tissue surrounding a lesion (SUR). In all animals, at least ten fields of view were measured using a 40 × objective lens. More than 500 LPCAT1-positive AEC2 per individual were measured for Ki67 and 1000 LPCAT1-positive AEC2 per individual were measured for γ-H2AX, and the mean value per individual was used for statistical analysis.

For the Tm4sf1 positive index in PDF and Agglomeration lesions, 50 PDF and 50 Agglomeration lesions were randomly selected from each 50 mg/m^3^ exposure group of each sex, and the percentage of TTF1/Tm4sf1 double positive AEP and TTF1-single positive AEC2 were measured.

### Statistical analysis

Except for the incidence and integrity of histopathological lesions, the data comparisons among multiple groups were performed as follows: when homogeneous variance and normal distribution were observed in samples without sex differences, a one-way ANOVA was used to compare the exposure and control groups. When the one-way ANOVA was significant, Dunnett’s multiple comparisons test was used to compare the control and exposure groups. If variances were significantly different, the control and exposure groups were evaluated using Kruskal–Wallis non-parametric analysis of variance. If the Kruskal–Wallis analysis was significant, the control and exposure groups were compared using Dunn’s test. The samples with sex differences were analyzed by two-way ANOVA with Tukey’s multiple comparison test. All statistical analyses were using performed GraphPad Prism 5 (GraphPad Software). The incidences and integrity of lesions were analyzed by the chi-square test using GraphPad Prism 5 (GraphPad Software). All statistical significance was set at *p* < 0.05.

## Supplementary Information

**Additional file 1: Fig. S1**. The whole body inhalation exposure system using in this study. The whole body inhalation exposure system (A). The averaged TiO_2_ NP concentration in the chamber for each exposure day (B). Representative scanning electron microscope (SEM) images of the TiO_2_ NPs in the chambers (C). The particle size distributions for the various exposure concentrations (D). Cumulative frequency distribution graphs with logarithmic probability (E). Mass median aerodynamic diameter (MMAD) and geometric standard deviation (σg) in the chamber (F). Scale bar: 20 μm, Yellow scale bar: 4 μm (panel C).

**Additional file 2: Fig. S2.** Biochemical markers in the BALF obtained from the lungs of rats after inhalation of TiO_2_ NPs for 13 weeks. Alkaline phosphatase (ALP) activity (A, B) and γ-Glutamyl transpeptidase (γ-GTP) activity (C, D) were measured using an automatic analyzer, and are shown by sex (males: A and C; females: B and D) (n=5). Statistical significance was analyzed using Dunn’s or Dunnett’s multiple comparison test: *p<0.05 and **p<0.01.

**Additional file 3:  Fig. S3**. Representative macroscopic photographs of whole lungs. A: Normal lungs of a female rat (0 mg/m^3^). B: TiO_2_ NPs exposed lungs of a female rat (50 mg/m^3^). Scale bar: 1 mm.

**Additional file 4:  Fig. S4.** Representative microscopic photographs of a female rat left lung after inhalation exposure to TiO_2_ NPs (50 mg/m^3^): same rat as shown in figure 8. The left lung was not injected with formalin through the bronchus into the lung, and formalin immersion fixation was performed after the lung was removed. A typical loupe image (A) of the entire left lung and magnified images of each lesion (B-D). Particles in the process of being eliminated by the mucociliary escalator were observed on the bronchial mucosa (B). The infiltration of naked TiO_2_ NPs or particle-laden macrophages in bronchus-associated lymphoid tissue (BALT) (C). Burst macrophages were scattered in the 50 mg/m^3^ group of both sexes (D).

**Additional file 5:  Fig. S5**. Representative microscopic photographs of the lungs of a female control rat. The lungs were stained with hematoxylin and eosin (HE) (see the Fig. 8 legend for details). A typical loupe image (A) of the entire lungs and magnified images of normal alveolar regions (B and C) are shown.

**Additional file 6: Fig. S6**. Representative microscopic photographs of mediastinal lymph nodes. A typical loupe image of the entire mediastinal lymph node and magnified images of each lymph node from a female control rat (A) and a female rat exposed to 50 mg/m^3^ (B) are shown.

**Additional file 7:  Fig. S7**. Immunohistochemistrical characteristics of bronchial lineage markers in tissue surrounding a lesion and in pulmonary dust foci. Representative immunohistochemical staining images of the club cell marker club cell secretory protein (CCSP), neuroendocrine cell marker calcitonin gene-related peptide (CGRP), basal cell marker p63, and bronchial epithelial lineage marker SRY-Box Transcription Factor 2 (Sox2) in the bronchus-bronchiole (A) and in pulmonary dust foci (B).

**Additional file 8: Fig. S8.** Additional AEC2 marker expression in pulmonary dust foci (PDF) and in tissue surrounding a lesion in a rat lung after inhalation exposure to TiO_2_ NP (50 mg/m^3^). Representative images of alveolar epithelial type 2 cell (AEC2) markers ABCA3 and proSPC in PDF and tissue surrounding a lesion.

**Additional file 9:  Fig. S9**. Immunohistochemical characteristics in tissue surrounding a lesion in rat lungs after inhalation exposure to TiO_2_ NP (50 mg/m^3^): same rat as in figure 9. Representative images of staining sets similar to figure 9 in tissue surrounding a lesion (normal tissue).

**Additional file 10:  Fig. S10**. Representative microscopic photographs of Masson’s trichrome and EVG staining in pulmonary dust foci and a pulmonary artery. Both stains were strongly positive in the arterial wall within the lung (right), but negative in the interstitium of the pulmonary dust foci (left). Abbreviations: EVG, Elastica Van Gieson.

**Additional file 11: Fig. S11**. Representative macroscopic and TEM images of TiO_2_ NP. A: Macroscopic image. B; TEM image.

**Additional file 12:  Fig. S12**. Design of the animal experimental protocol used in this study.

**Additional file 13: Table S1**. Numerical values of the Means and SD data in Figures 1, 3, 4, 5, 6, and S2.

**Additional file 14: Table S2**. Absolute organ weights in the 13-week inhalation exposure study.

**Additional file 15: Table S3**. Relative organ weights in the 13-week inhalation exposure study.

**Additional file 16: Table S4.** Blood-hematologic data in the 13-week inhalation exposure study.

**Additional file 17: Table S5.**. Blood-biochemistry data in the 13-week inhalation exposure study.

**Additional file 18: Table S6**. Histopathological findings, excluding lung and mediastinal lymph node, in the 13-week inhalation exposure study.

**Additional file 19: Table S7**. Calculation of benchmark doses (BMD) for pulmonary dust foci (PDF) using EPA's Benchmark Dose Software (BMDS 3.2)

**Additional file 20: Table S8**. Calculation of the internal lung dose using the MPPD particle deposition model ver. 3.04.

**Additional file 21: Table S9**. Calculation of human inhalation exposure concentrations by conversion from benchmark dose lower confidence limits for rat PDF lesions.

**Additional file 22: Table S10**. Summary of the effect of inhalation exposure to TiO_2_ (the present study) and ultrafine TiO_2_ particles (P25) on LDH activity.

**Additional file 23: Table S11**. Characteristics of TiO_2_ NP used in this study.

**Additional file 24: Table S12**. List of primary antibodies used in this study.

## Data Availability

The datasets used and analyzed during the current study are available from the corresponding authors on reasonable request.

## Abbreviations

ABCA3: ATP-binding cassette transporter3
AEC1: Alveolar epithelial type 1 cell
AEC2: Alveolar epithelial type 2 cell
AEP: Alveolar epithelial progenitor
ALP: Alkaline phosphatase
AM: Alveolar macrophage
αSMA: α-Smooth muscle actin
BALF: Bronchoalveolar lavage fluid
BALT: Bronchus-associated lymphoid tissue
CCSP: Club cell secretory protein
CGRP: Calcitonin gene-related peptide
σg: Geometric standard deviations
γ-GTP: γ-Glutamyl transpeptidase
γ-H2AX: Phosphorylation of the Ser-139 residue of the histone variant H2AX
HE: Hematoxylin and eosin
INHAND: International Harmonization of Nomenclature and Diagnostic Criteria for Lesions in Rats and Mice
LDH: Lactate dehydrogenase
LOAEC: Lowest observed adverse effect concentration
LPCAT1: Lysophosphatidylcholine acyltransferase 1
MMAD: Mass median aerodynamic diameter
NOAEC: No observed adverse effect concentration
PDF: Pulmonary dust foci
proSPC: Prosurfactant protein C
SEM: Scanning electron microscope
Sox2: SRY-Box Transcription Factor 2
SUR: Tissue surrounding a lesion
TiO_2_ NPs: Titanium dioxide nanoparticles
Tm4sf1: Transmembrane 4 superfamily member 1
TTF1: Thyroid Transcription Factor 1
VEGFR3: Vascular endothelial growth factor receptor 3
