# Supplementary material for: Pulmonary dust foci as rat pneumoconiosis lesion induced by titanium dioxide nanoparticles in 13-week inhalation study
Source: Part Fibre Toxicol. 2022 Sep 14;19:58. doi: 10.1186/s12989-022-00498-3 (PMC9472424; doi:10.1186/s12989-022-00498-3)
Supplement: Supplementary file 1 — Additional file 1: Fig. S1. The whole body inhalation exposure system using in this study. The whole body inhalation exposure system (A). The averaged TiO2 NP concentration in the chamber for each exposure day (B). Representative scanning electron microscope (SEM) images of the TiO2 NPs in the chambers (C). The particle size distributions for the various exposure concentrations (D). Cumulative frequency distribution graphs with logarithmic probability (E). Mass median aerodynamic diameter (MMAD) and geometric standard deviation (σg) in the chamber (F). Scale bar: 20 μm, Yellow scale bar: 4 μm (panel C). [file 12989_2022_498_MOESM1_ESM.pdf]

Fig. S1

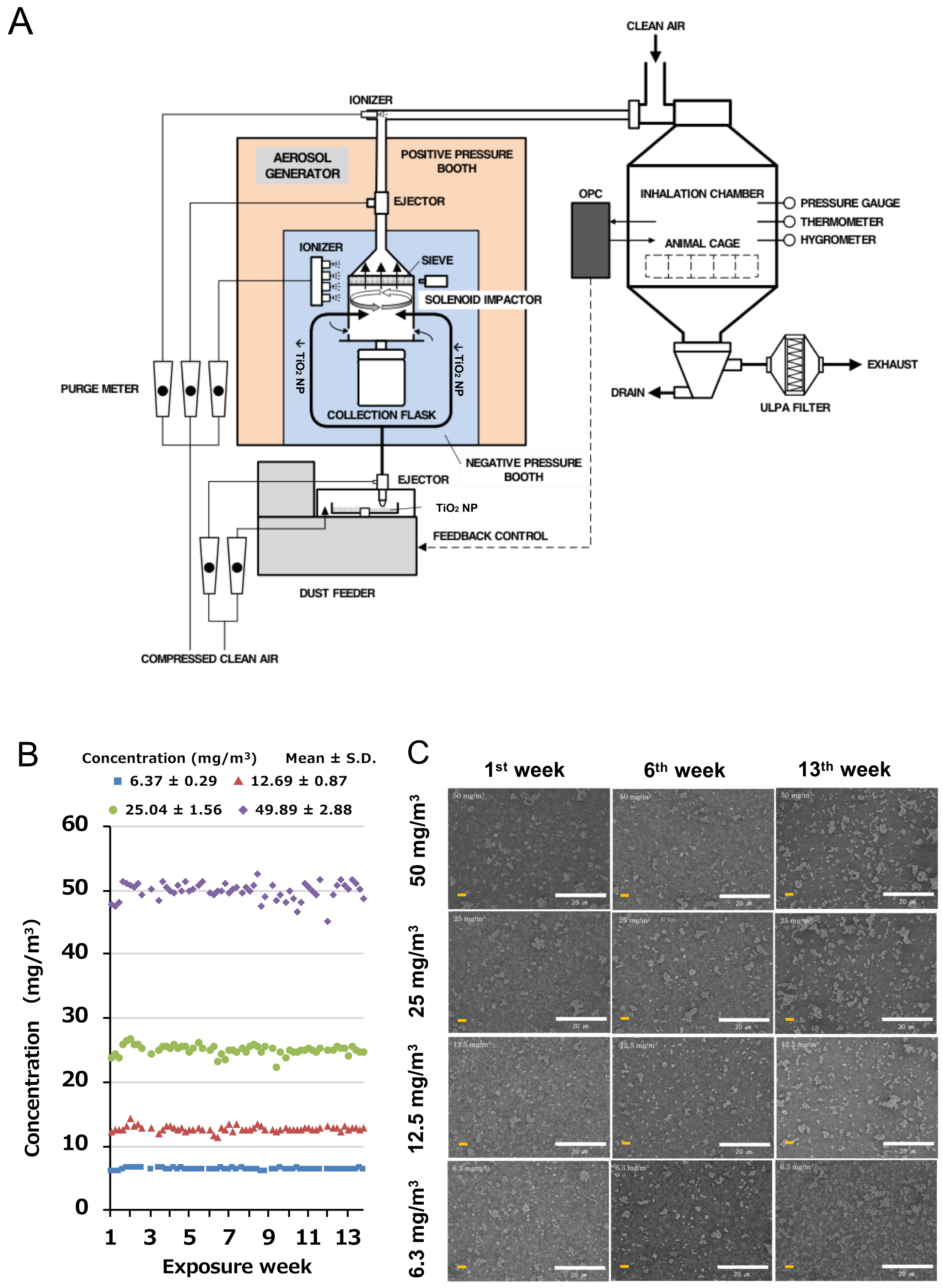
A
CLEAN AIR
IONIZER
AEROSOL GENERATOR
POSITIVE PRESSURE BOOTH
EJECTOR
IONIZER
SIEVE
SOLENOID IMPACTOR
TiO2 NP
TiO2 NP
COLLECTION FLASK
PURGE METER
NEGATIVE PRESSURE BOOTH
EJECTOR
TiO2 NP
FEEDBACK CONTROL
DUST FEEDER
COMPRESSED CLEAN AIR
OPC
INHALATION CHAMBER
ANIMAL CAGE
PRESSURE GAUGE
THERMOMETER
HYGROMETER
DRAIN
EXHAUST
ULPA FILTER
B
Concentration (mg/m3) Mean ± S.D.
6.37 ± 0.29
12.69 ± 0.87
25.04 ± 1.56
49.89 ± 2.88
Concentration (mg/m3)
0
10
20
30
40
50
60
1
3
5
7
9
11
13
Exposure week
C
1st week
6th week
13th week
50 mg/m3
25 mg/m3
12.5 mg/m3
6.3 mg/m3

Fig. S1

D

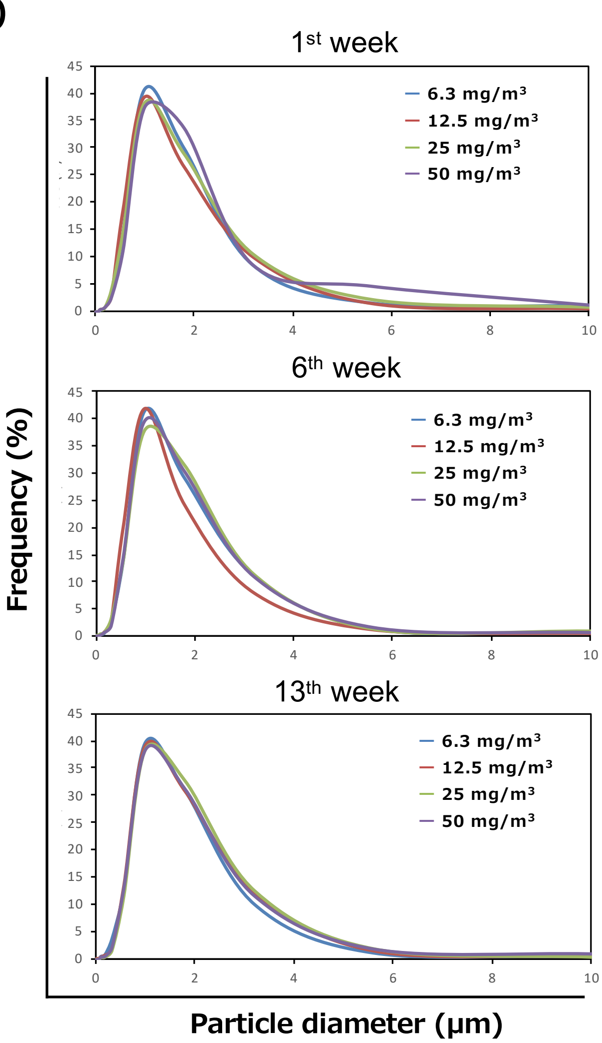

E

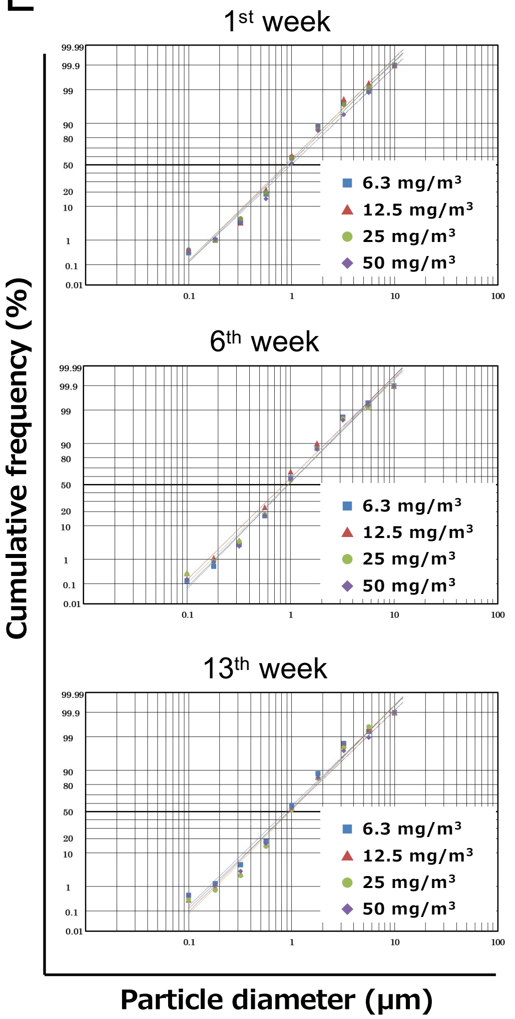

F

Experimental weeks	6.3 mg/m^3		12.5 mg/m^3		25 mg/m^3		50 mg/m^3	
	MMAD (μm)	σg	MMAD (μm)	σg	MMAD (μm)	σg	MMAD (μm)	σg
1st exposure	0.9	2.1	0.9	2.1	0.9	2.1	1.0	2.1
6th exposure	1.0	2.0	0.9	2.1	0.9	2.1	1.0	2.0
13th exposure	0.9	2.1	0.9	2.0	1.0	2.0	0.9	2.1

Fig. S2

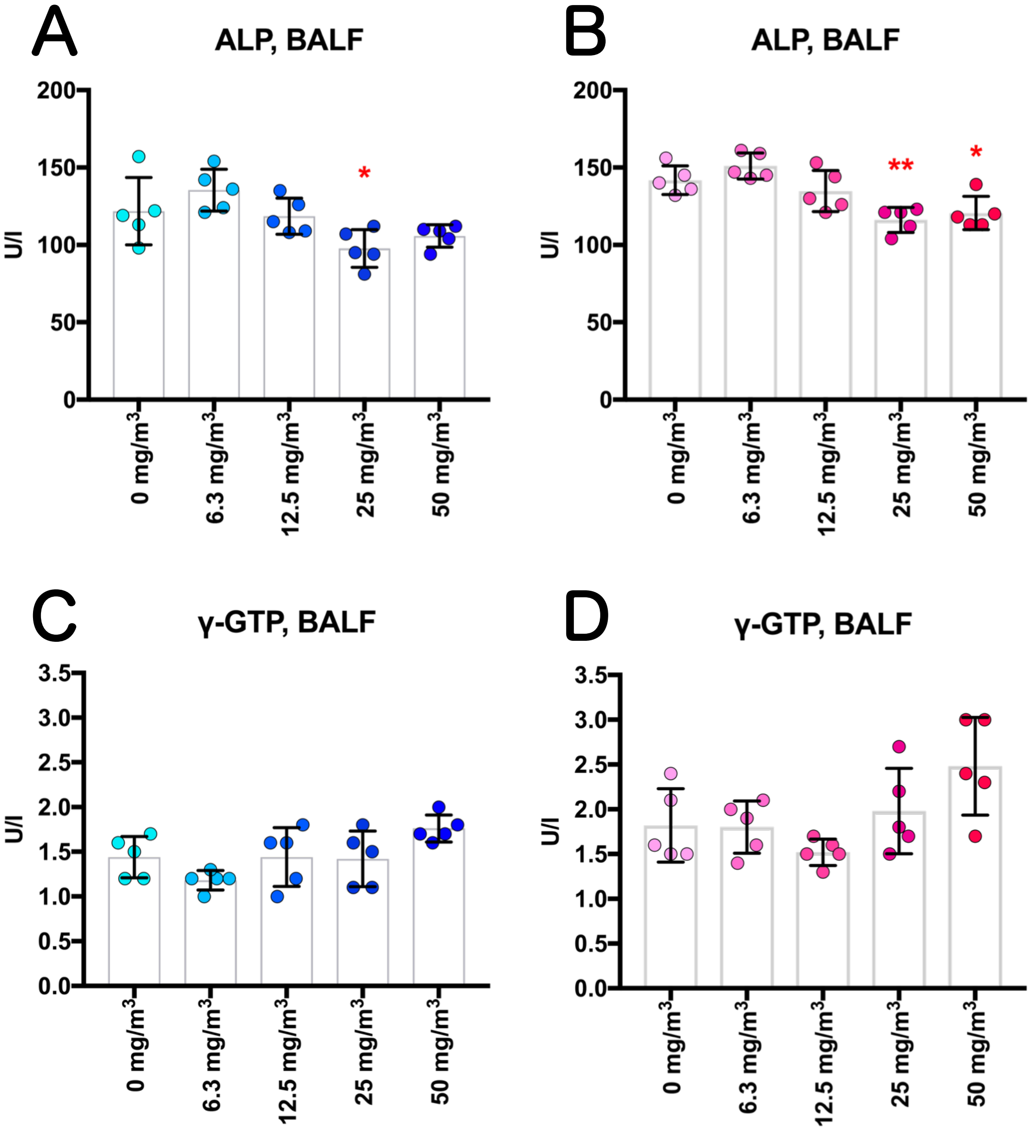

A
ALP, BALF
B
ALP, BALF
C
γ-GTP, BALF
D
γ-GTP, BALF
U/l
0
50
100
150
200
0.0
0.5
1.0
1.5
2.0
2.5
3.0
3.5
*
**
*
0 mg/m3
6.3 mg/m3
12.5 mg/m3
25 mg/m3
50 mg/m3

Fig. S3

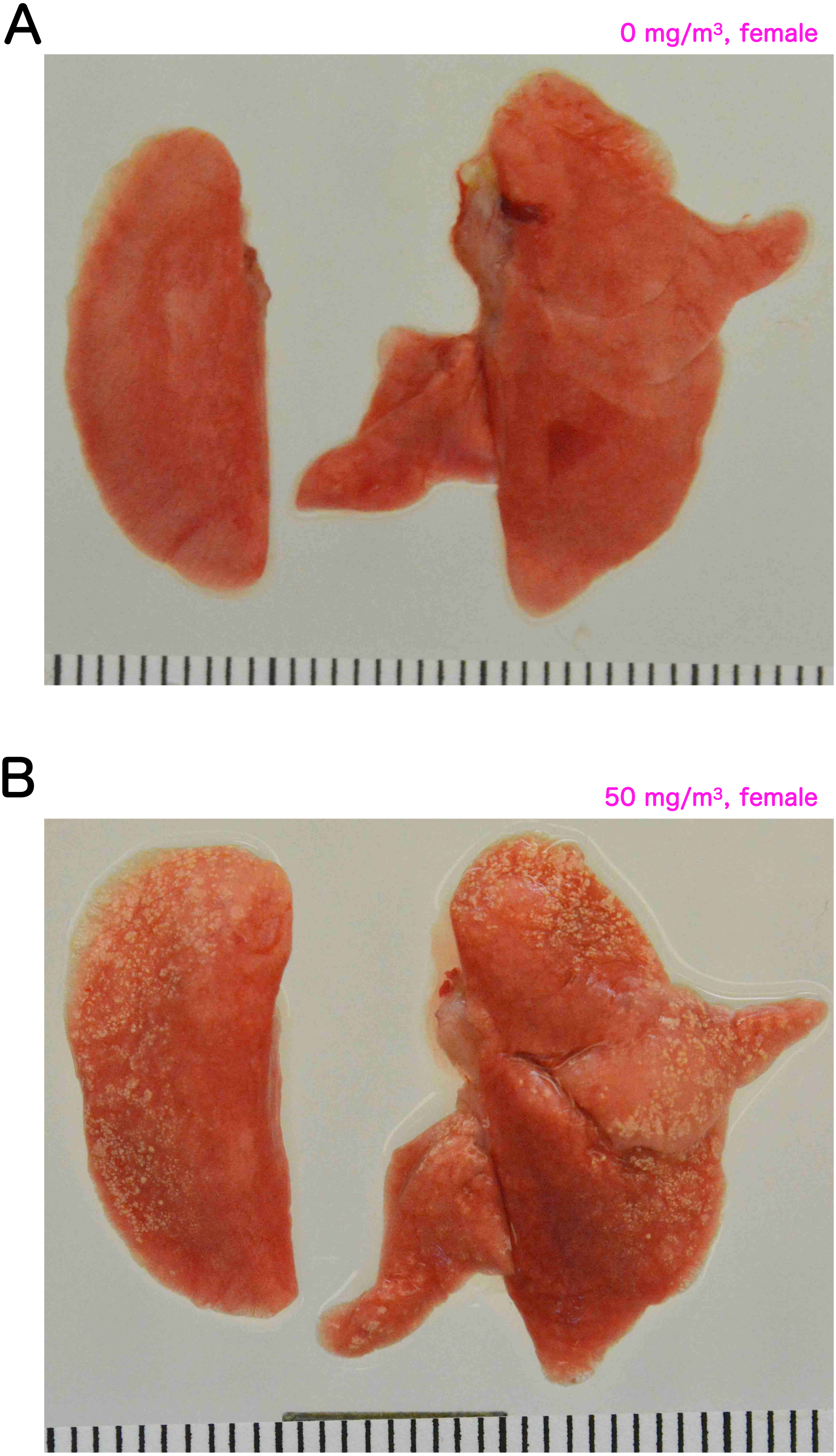

Fig. S4

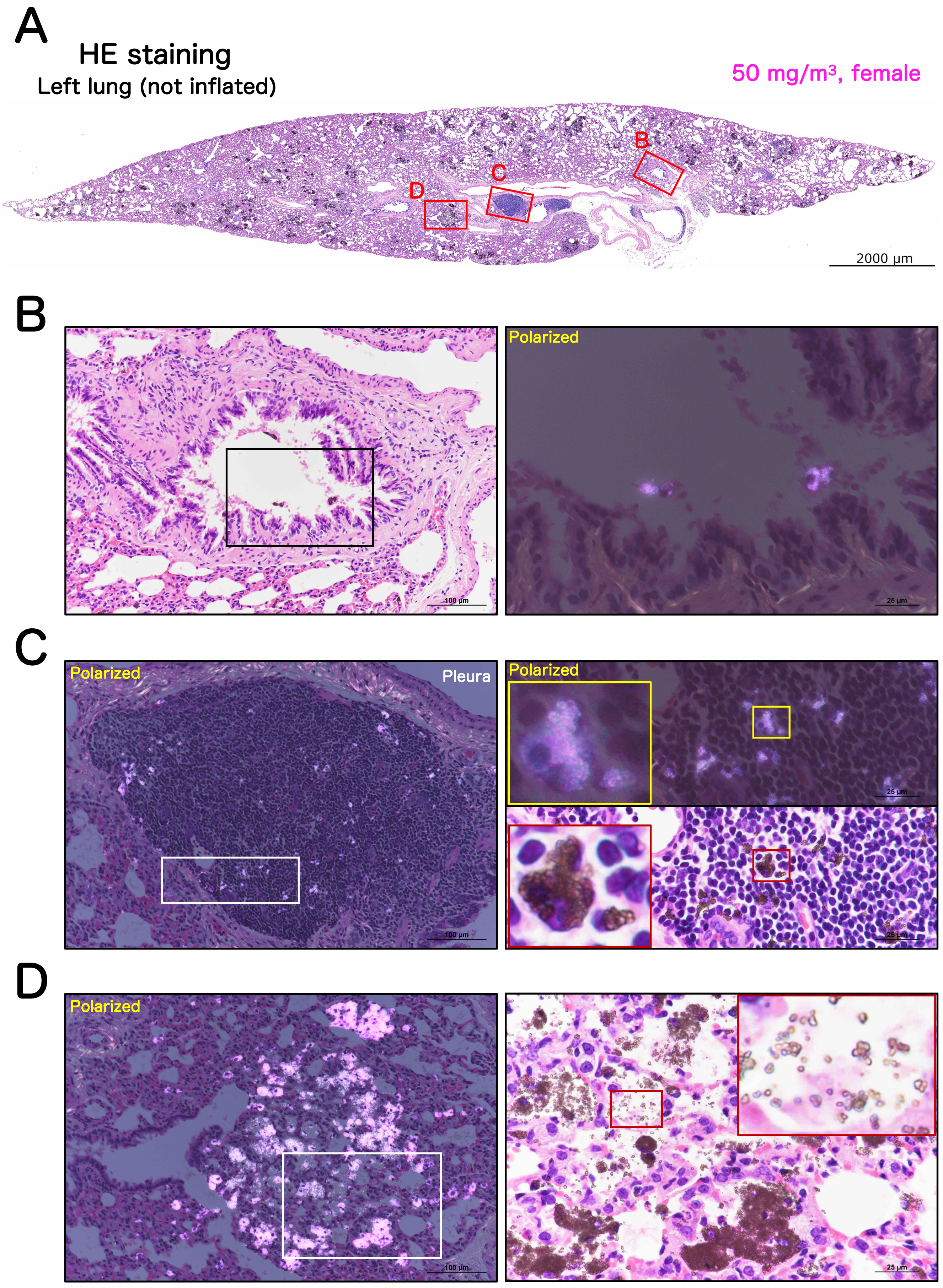

A
HE staining
Left lung (not inflated)
50 mg/m3, female
B
C
D
2000 μm
B
Polarized
100 μm
25 μm
C
Polarized
Pleura
100 μm
Polarized
25 μm
25 μm
D
Polarized
100 μm
25 μm

Fig. S5

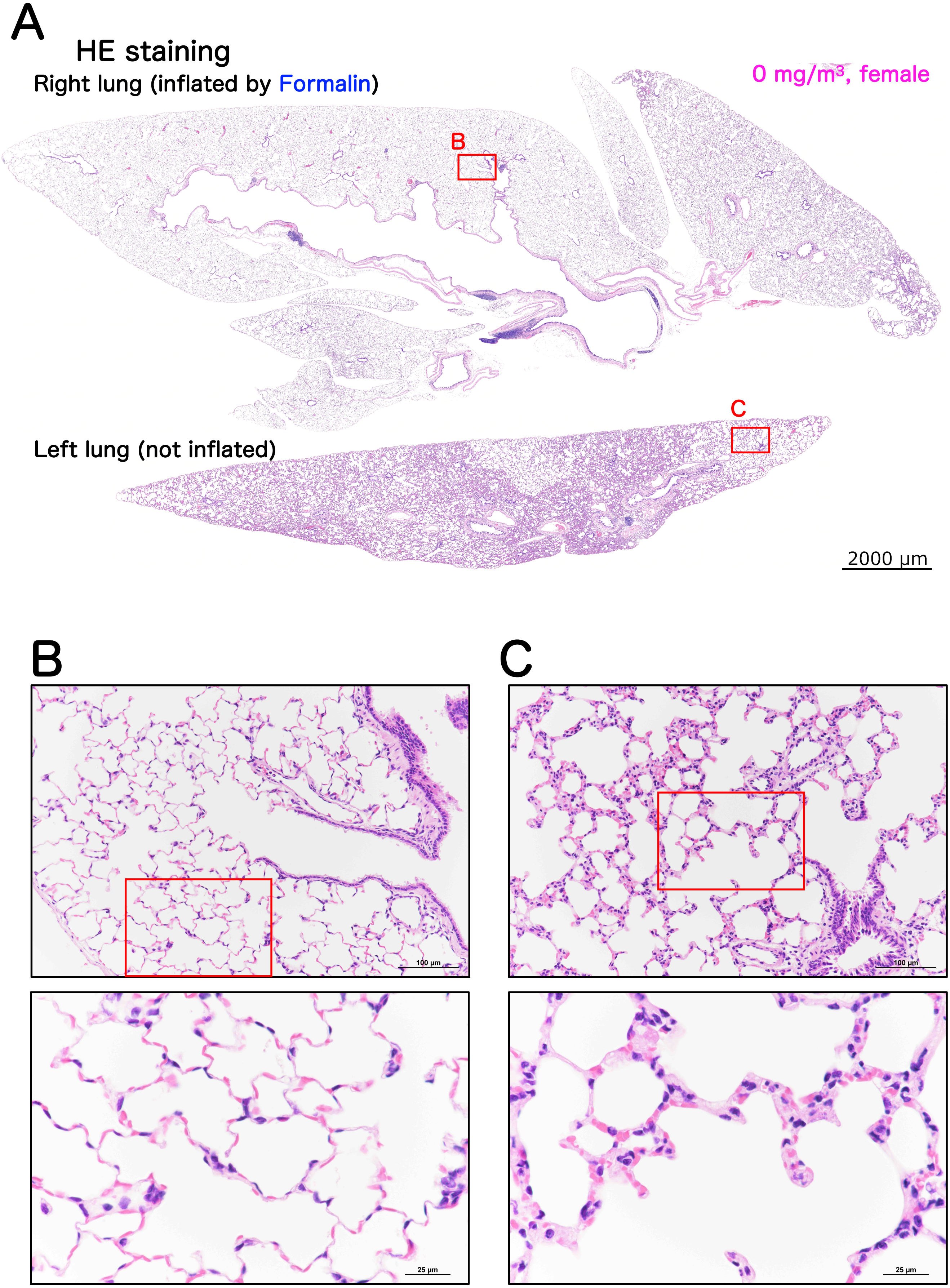
A
HE staining
Right lung (inflated by Formalin)
0 mg/m3, female
B
C
Left lung (not inflated)
2000 μm
B
C
100 μm
100 μm
25 μm
25 μm
