# Supplementary material for: Pulmonary dust foci as rat pneumoconiosis lesion induced by titanium dioxide nanoparticles in 13-week inhalation study
Source: Part Fibre Toxicol. 2022 Sep 14;19:58. doi: 10.1186/s12989-022-00498-3 (PMC9472424; doi:10.1186/s12989-022-00498-3)
Supplement: Supplementary file 2 — Additional file 2: Fig. S2. Biochemical markers in the BALF obtained from the lungs of rats after inhalation of TiO2 NPs for 13 weeks. Alkaline phosphatase (ALP) activity (A, B) and γ-Glutamyl transpeptidase (γ-GTP) activity (C, D) were measured using an automatic analyzer, and are shown by sex (males: A and C; females: B and D) (n=5). Statistical significance was analyzed using Dunn’s or Dunnett’s multiple comparison test: *p<0.05 and **p<0.01. [file 12989_2022_498_MOESM2_ESM.pdf]

Fig. S6

A

Mediastinal lymph node (0 mg/m³, female)

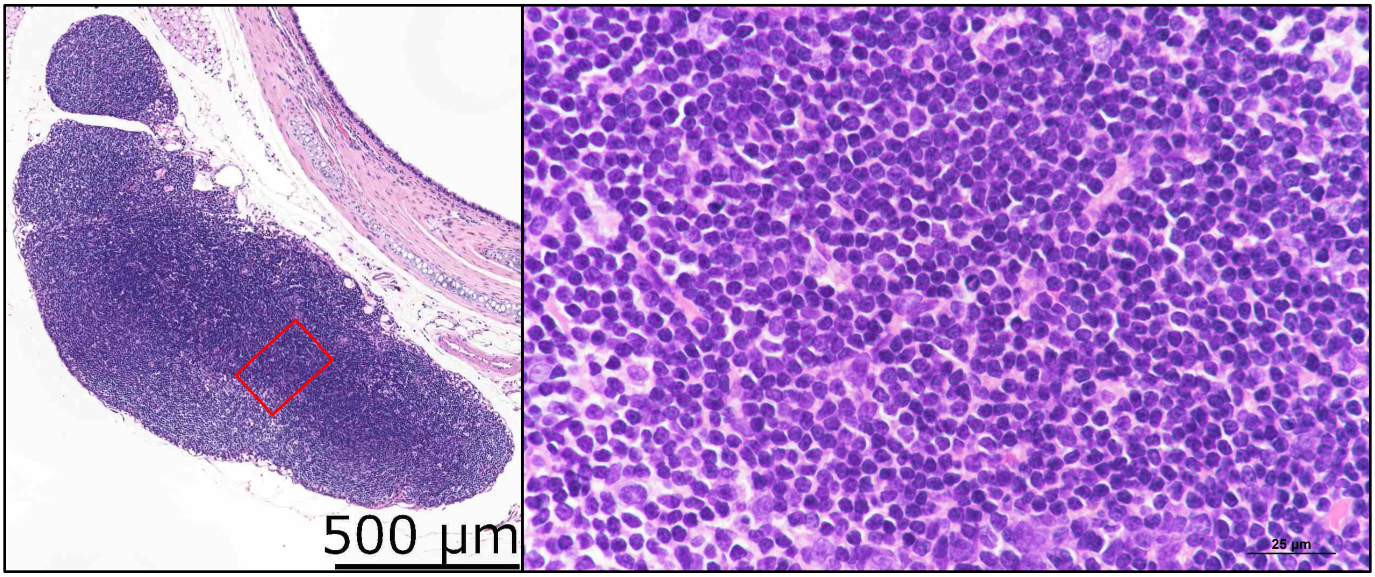

B

Mediastinal lymph node (50 mg/m³, female)

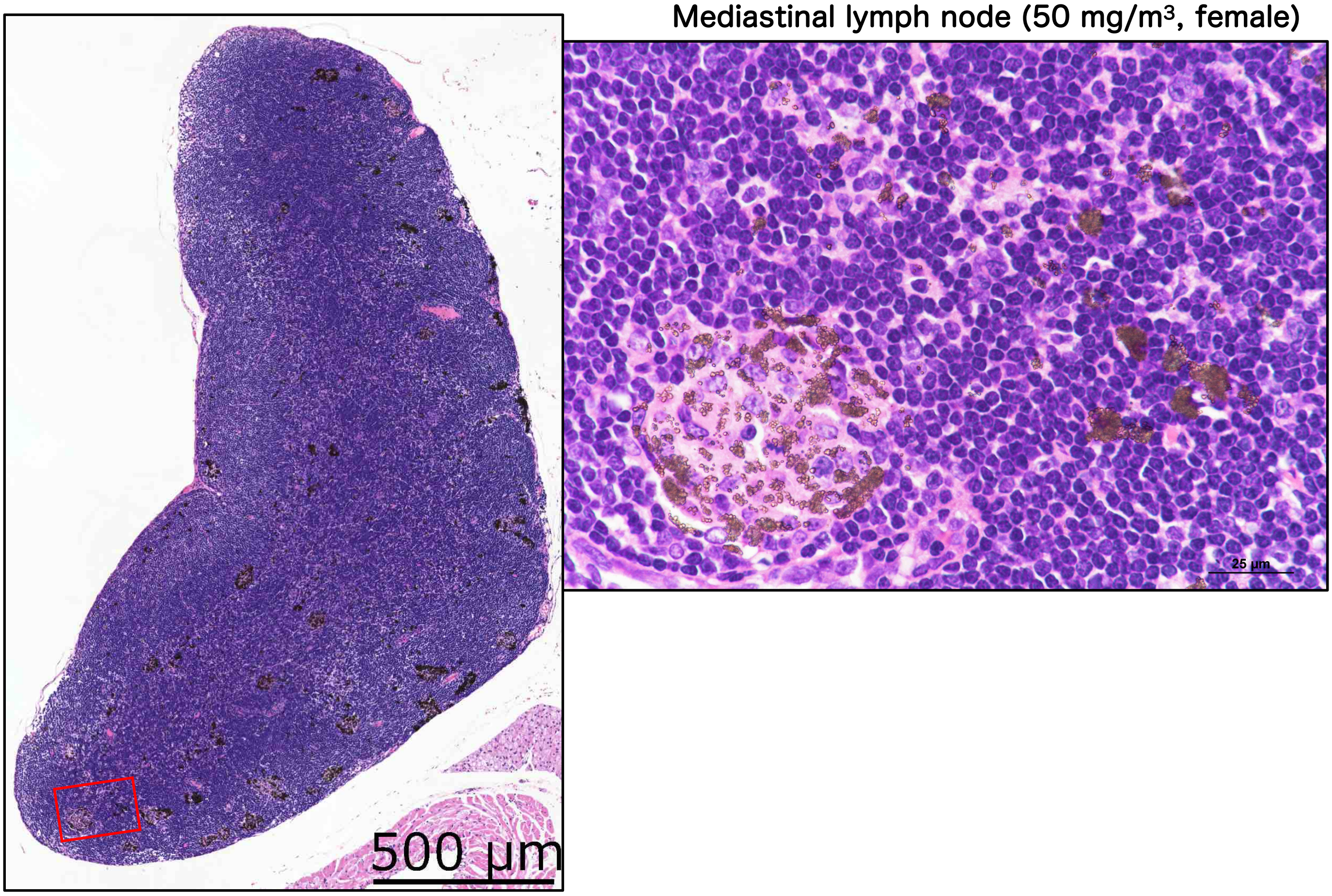

Fig. S7

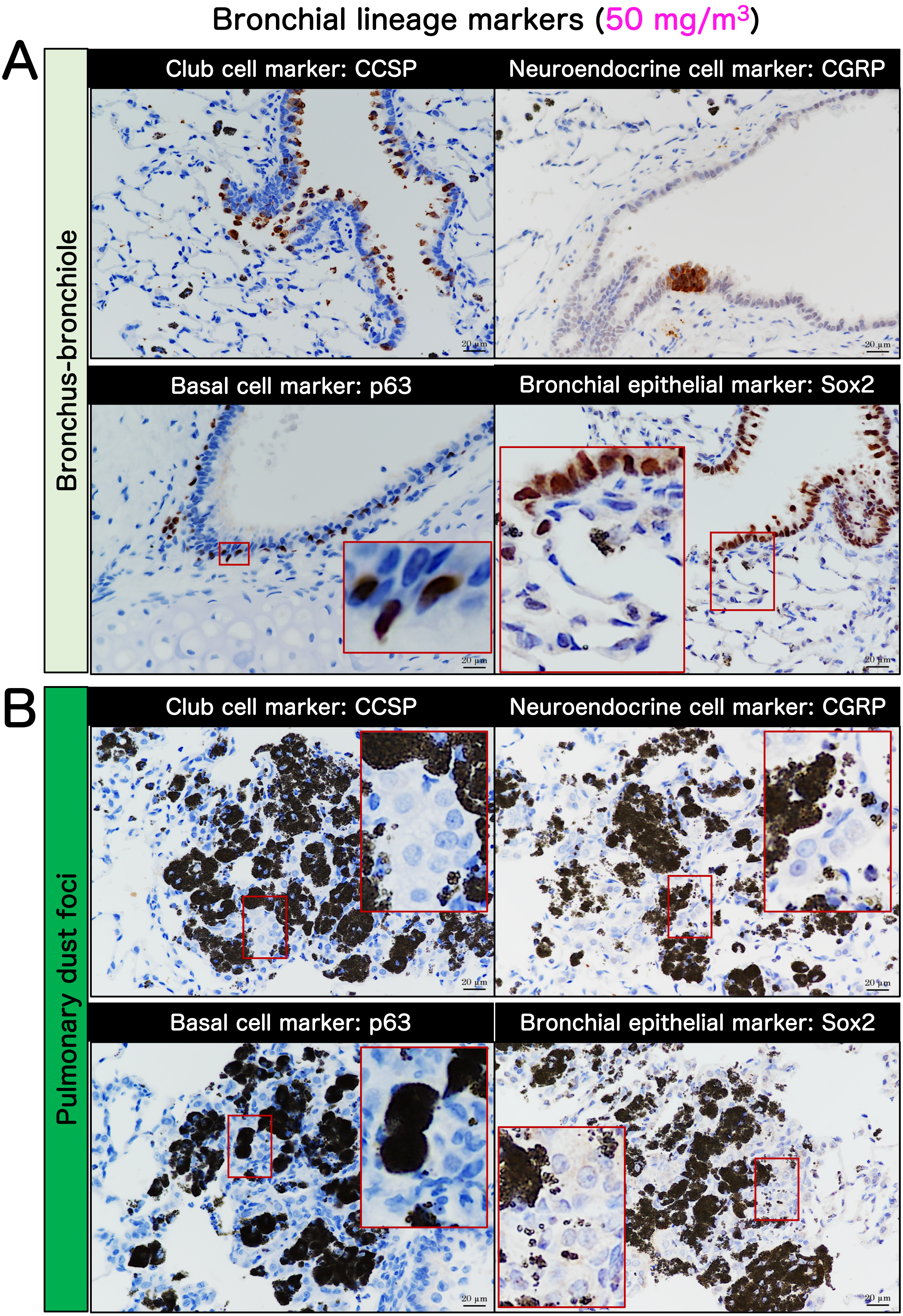

Bronchial lineage markers (50 mg/m3)
A
Bronchus-bronchiole
Club cell marker: CCSP
Neuroendocrine cell marker: CGRP
Basal cell marker: p63
Bronchial epithelial marker: Sox2
B
Pulmonary dust foci
Club cell marker: CCSP
Neuroendocrine cell marker: CGRP
Basal cell marker: p63
Bronchial epithelial marker: Sox2

Fig. S8

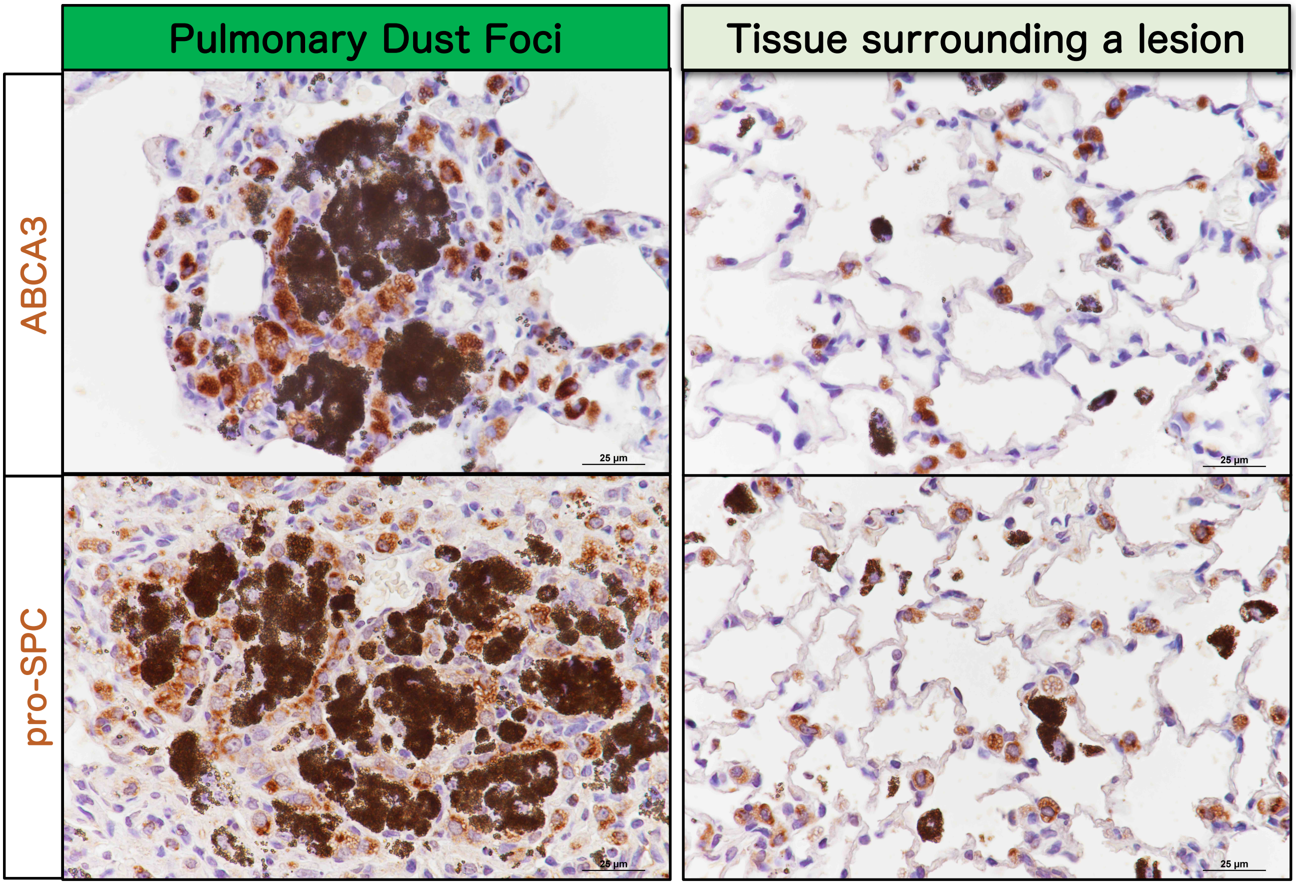

Pulmonary Dust Foci
Tissue surrounding a lesion
ABCA3
pro-SPC
25 µm
25 µm
25 µm
25 µm

Fig. S9

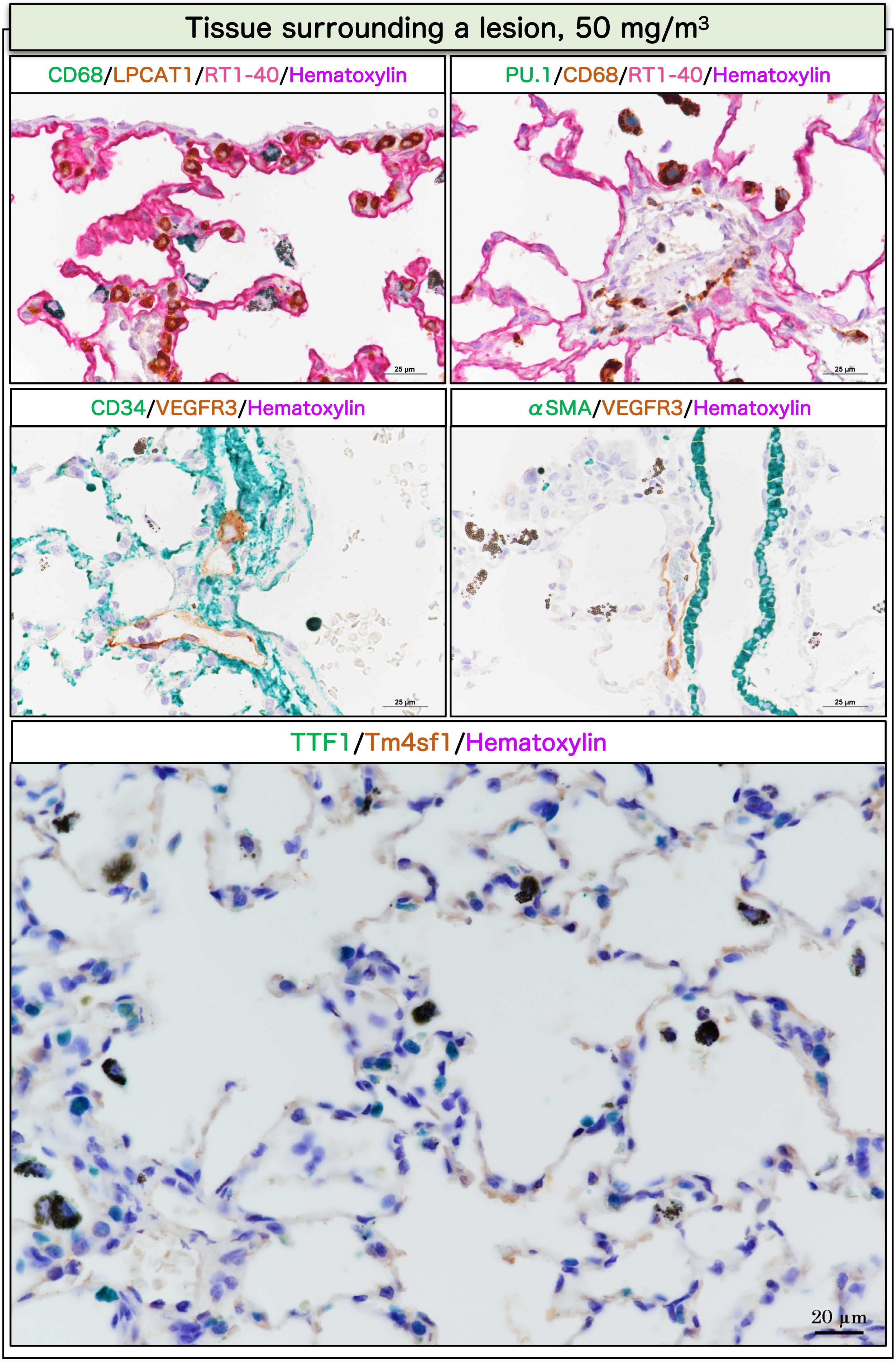

Tissue surrounding a lesion, 50 mg/m³
CD68/LPCAT1/RT1-40/Hematoxylin
PU.1/CD68/RT1-40/Hematoxylin
CD34/VEGFR3/Hematoxylin
αSMA/VEGFR3/Hematoxylin
TTF1/Tm4sf1/Hematoxylin
25 µm
20 µm

Fig. S10

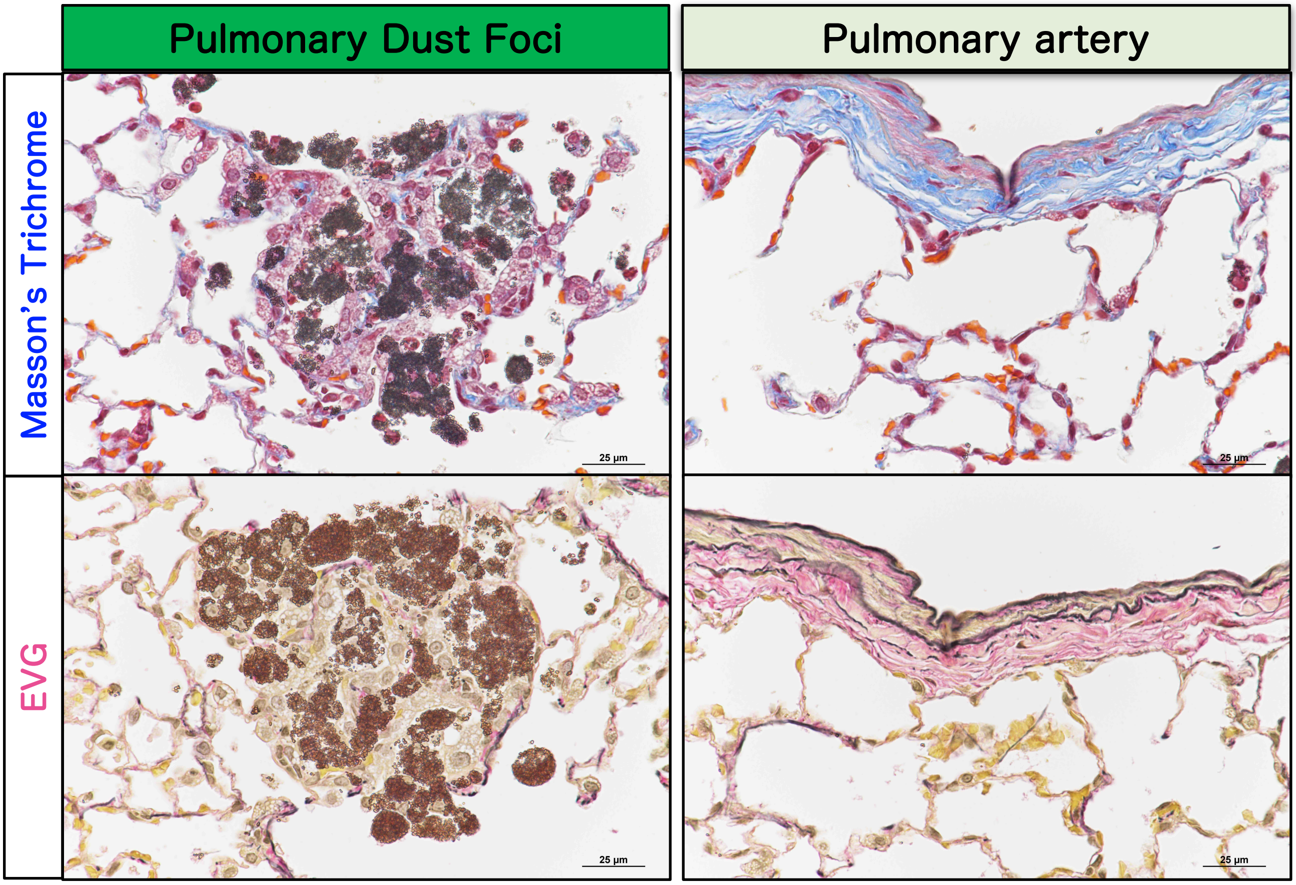
Pulmonary Dust Foci
Pulmonary artery
Masson's Trichrome
EVG
25 μm
25 μm
25 μm
25 μm

Fig. S11

A

B

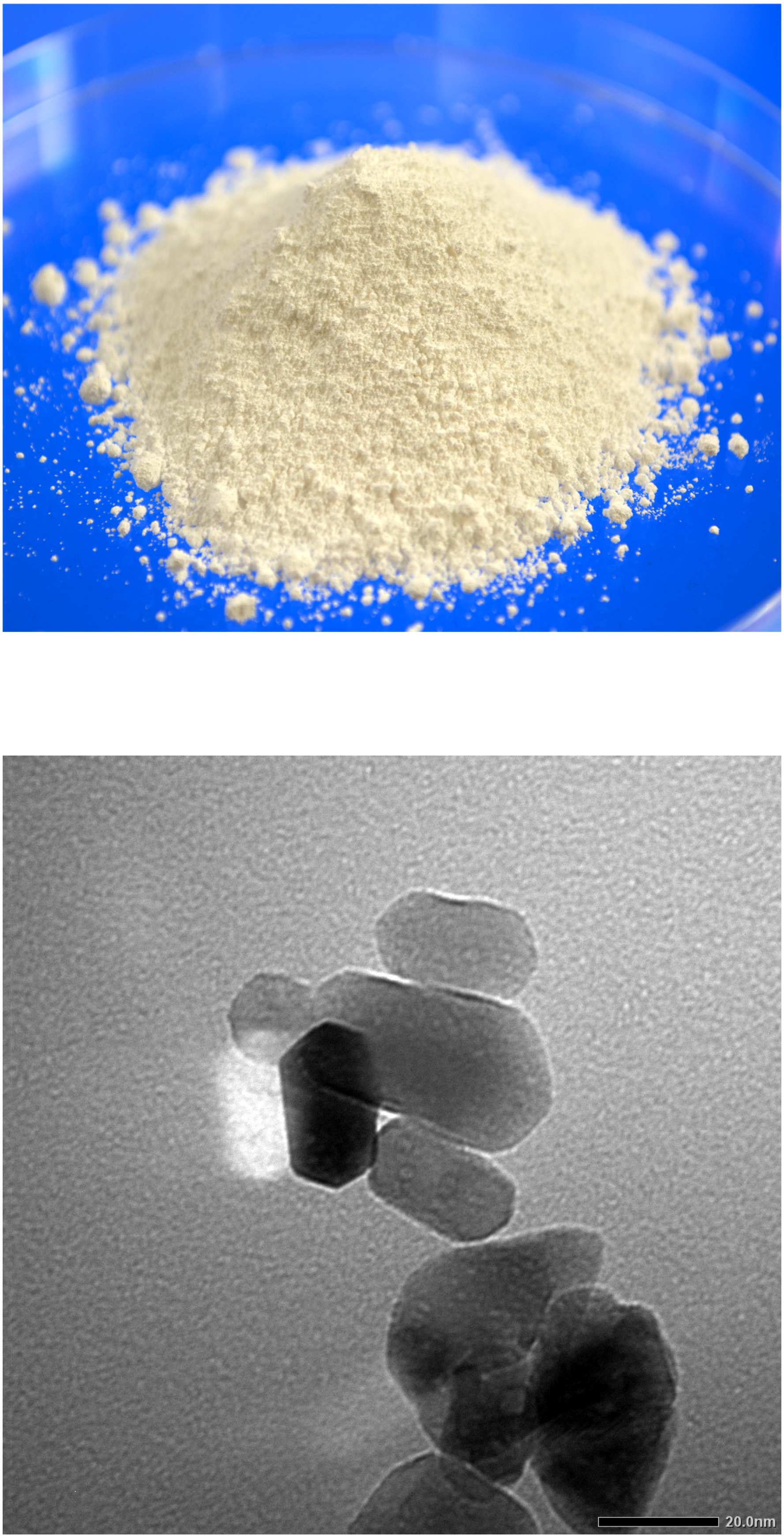

Fig. S12

Experimental protocol
(inhalation study: dose-response)

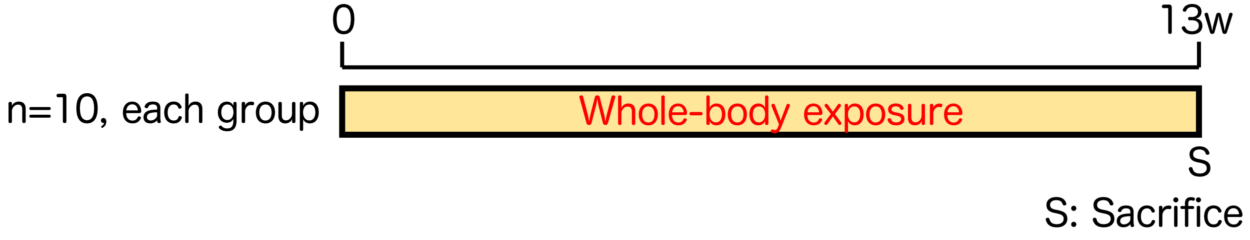

Animal: F344/DuCrlCrlj rat, 6-week-old
male (n=10 each group), female (n=10 each group), total n=100
Test compound: Anatase type titanium dioxide nanoparticles (primary particle size: 30 nm)
Exp. Conc.: 6hr/day, 5 day/week, 0, 6.3, 12.5 , 25 and 50 mg/m^3
