# Supplementary figures and images for: Pulmonary dust foci as rat pneumoconiosis lesion induced by titanium dioxide nanoparticles in 13-week inhalation study
Source: Part Fibre Toxicol. 2022 Sep 14;19:58. doi: 10.1186/s12989-022-00498-3 (PMC9472424; doi:10.1186/s12989-022-00498-3)

Fig. S3

A

0 mg/m<sup>3</sup>, female

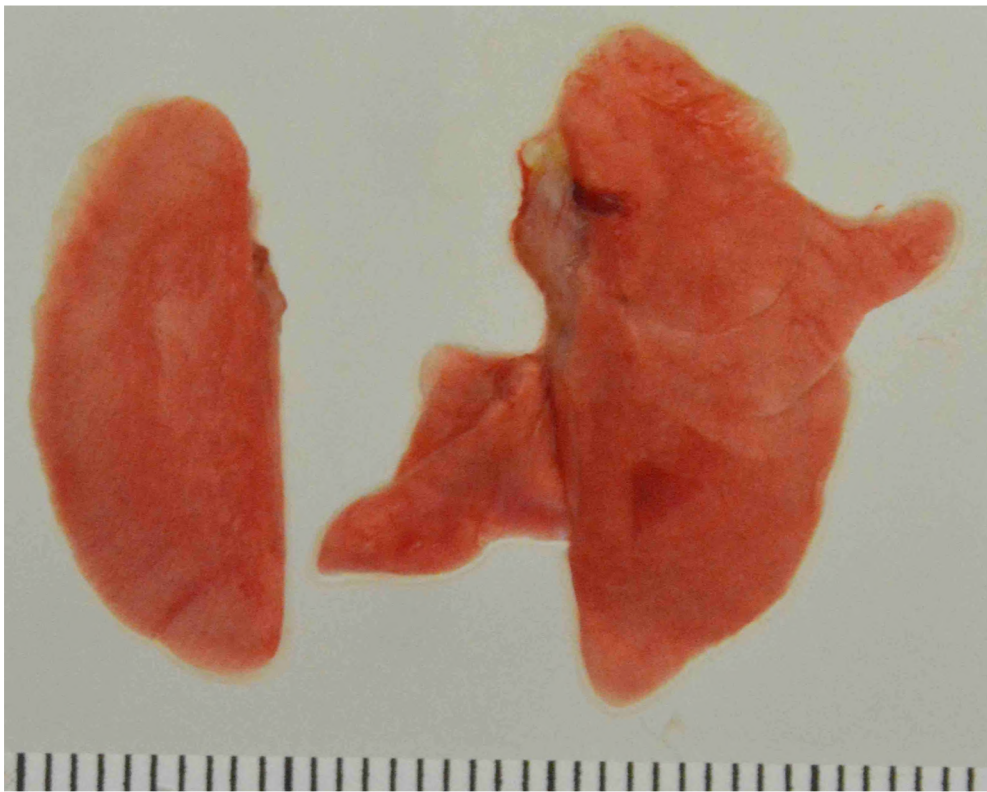

B

50 mg/m<sup>3</sup>, female

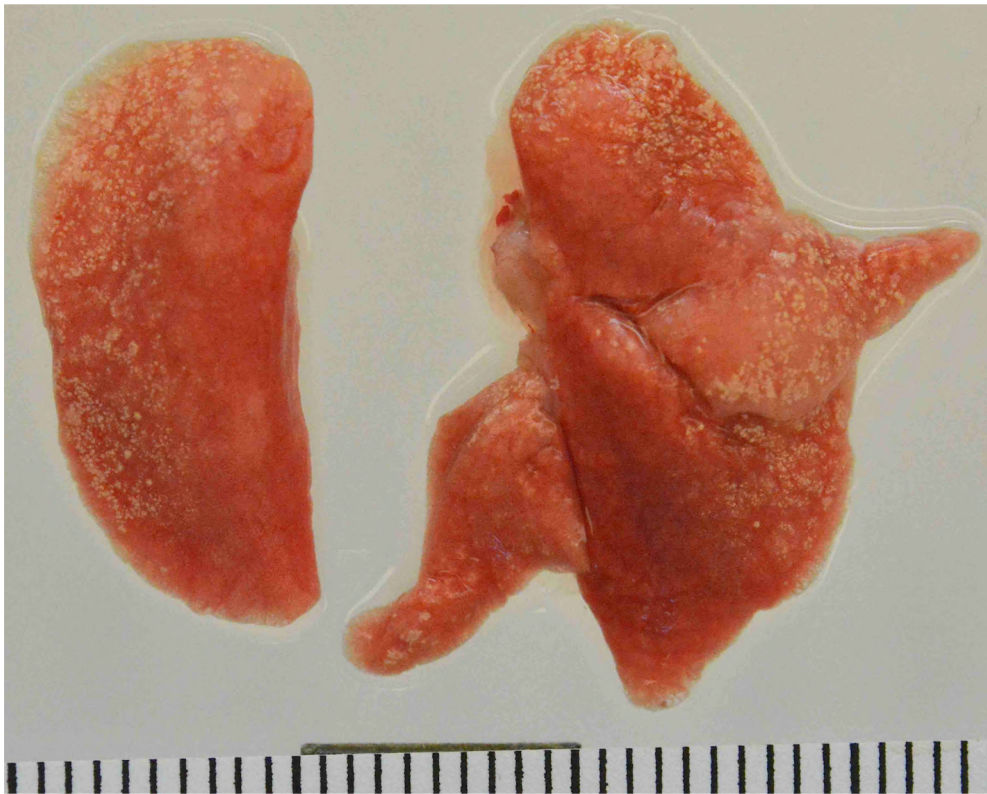

Supplement: Supplementary file 3 — Additional file 3: Fig. S3. Representative macroscopic photographs of whole lungs. A: Normal lungs of a female rat (0 mg/m3). B: TiO2 NPs exposed lungs of a female rat (50 mg/m3). Scale bar: 1 mm. [file 12989_2022_498_MOESM3_ESM.pdf]

Fig. S4

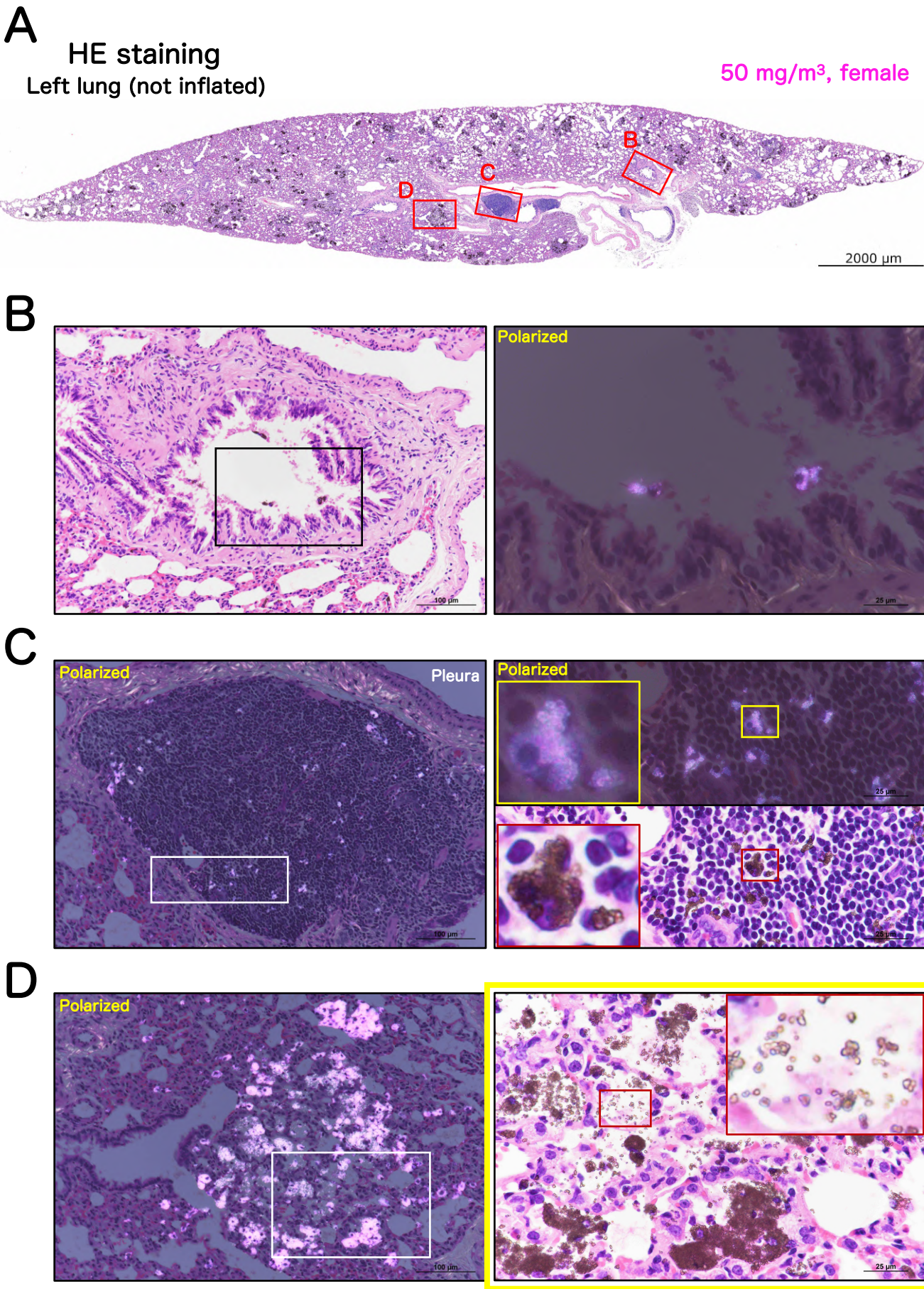

Supplement: Supplementary file 4 — Additional file 4: Fig. S4. Representative microscopic photographs of a female rat left lung after inhalation exposure to TiO2 NPs (50 mg/m3): same rat as shown in figure 8. The left lung was not injected with formalin through the bronchus into the lung, and formalin immersion fixation was performed after the lung was removed. A typical loupe image (A) of the entire left lung and magnified images of each lesion (B-D). Particles in the process of being eliminated by the mucociliary escalator were observed on the bronchial mucosa (B). The infiltration of naked TiO2 NPs or particle-laden macrophages in bronchus-associated lymphoid tissue (BALT) (C). Burst macrophages were scattered in the 50 mg/m3 group of both sexes (D). [file 12989_2022_498_MOESM4_ESM.pdf]

Fig. S5

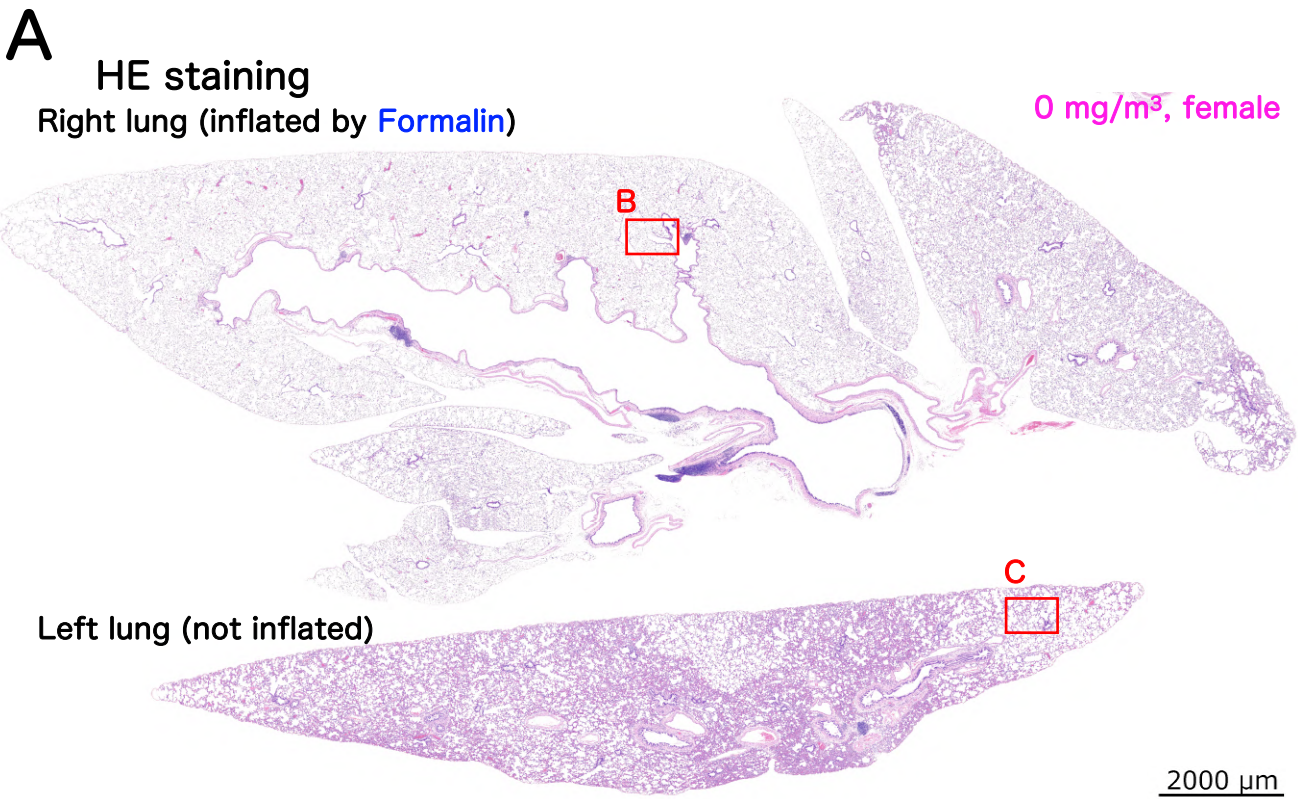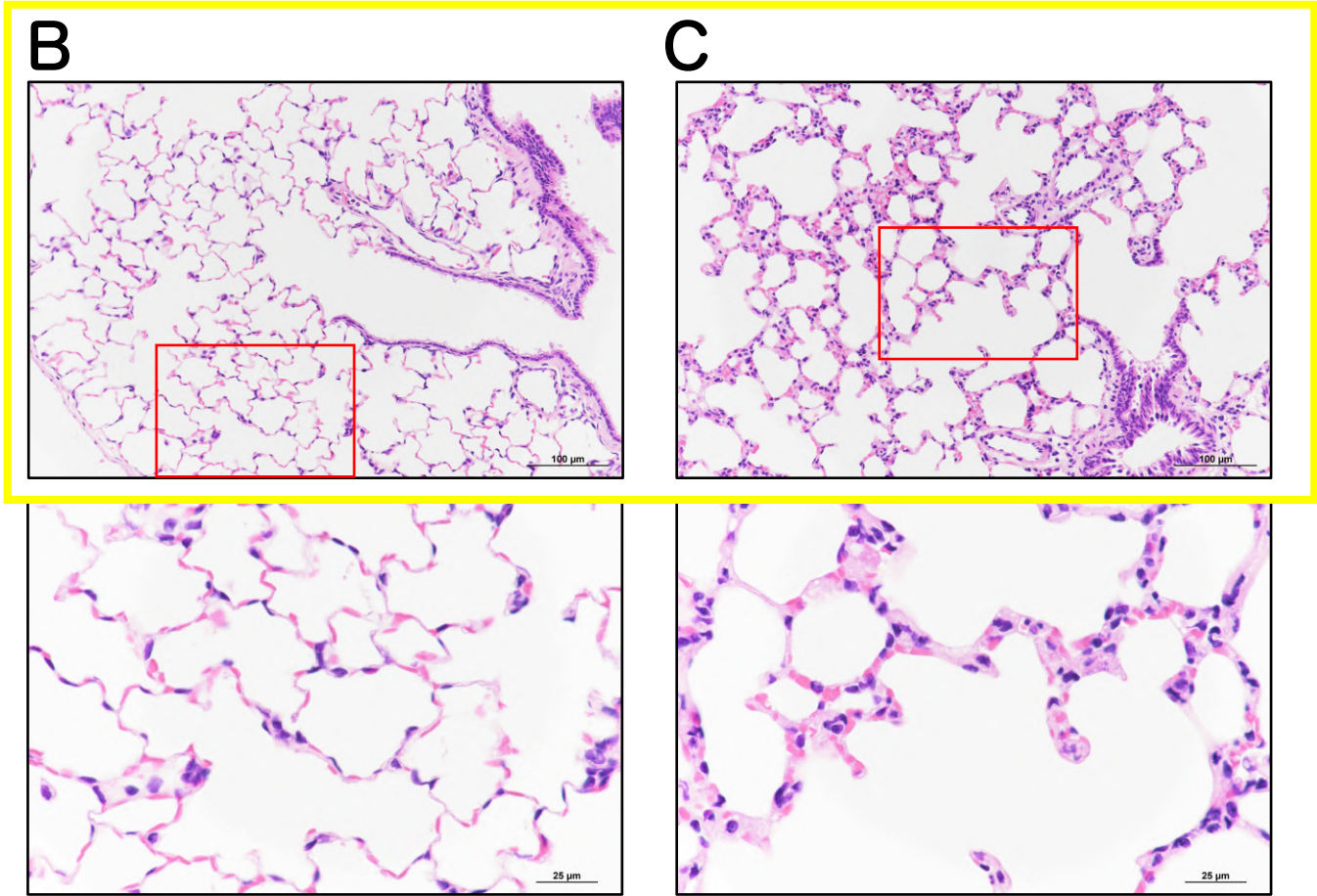

Supplement: Supplementary file 5 — Additional file 5: Fig. S5. Representative microscopic photographs of the lungs of a female control rat. The lungs were stained with hematoxylin and eosin (HE) (see the Fig. 8 legend for details). A typical loupe image (A) of the entire lungs and magnified images of normal alveolar regions (B and C) are shown. [file 12989_2022_498_MOESM5_ESM.pdf]

Fig. S6

A

Mediastinal lymph node (0 mg/m<sup>3</sup>, female)

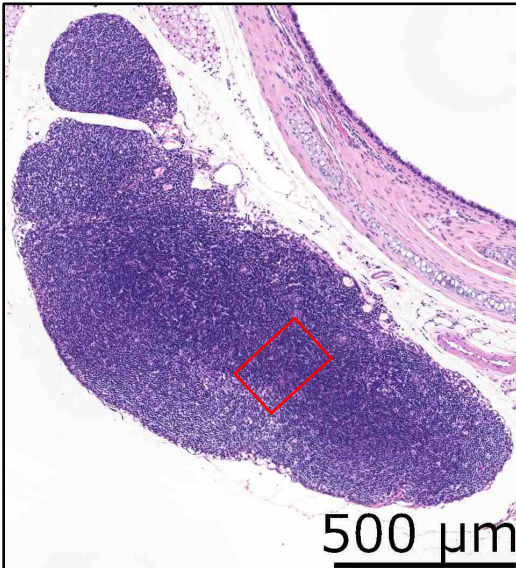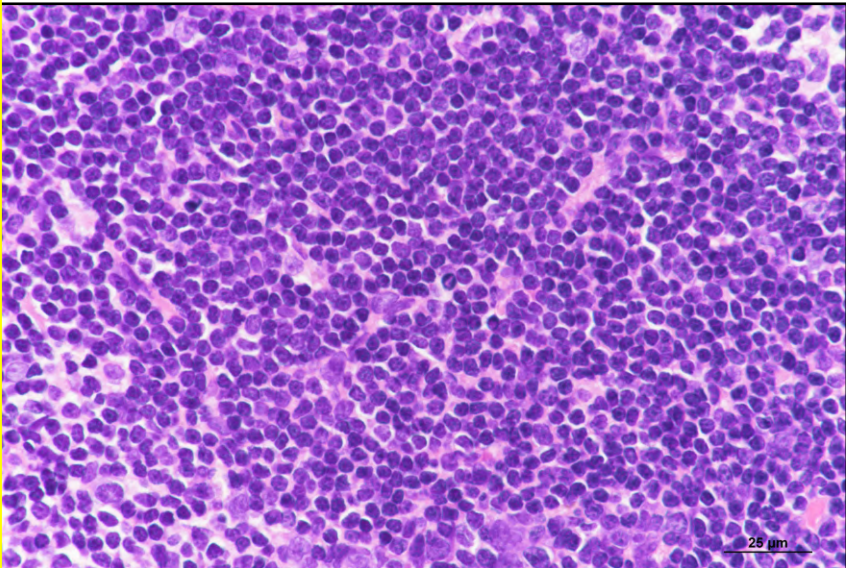

B

Mediastinal lymph node (50 mg/m<sup>3</sup>, female)

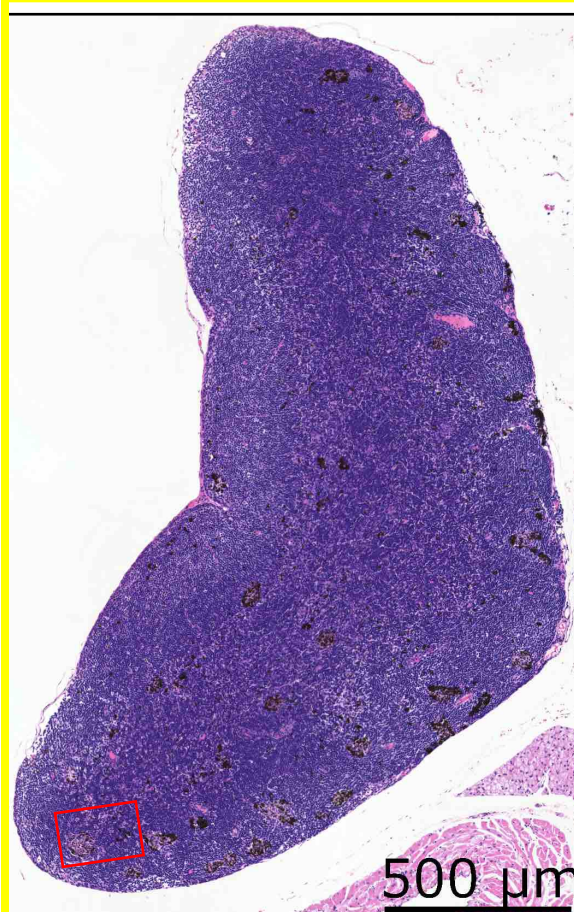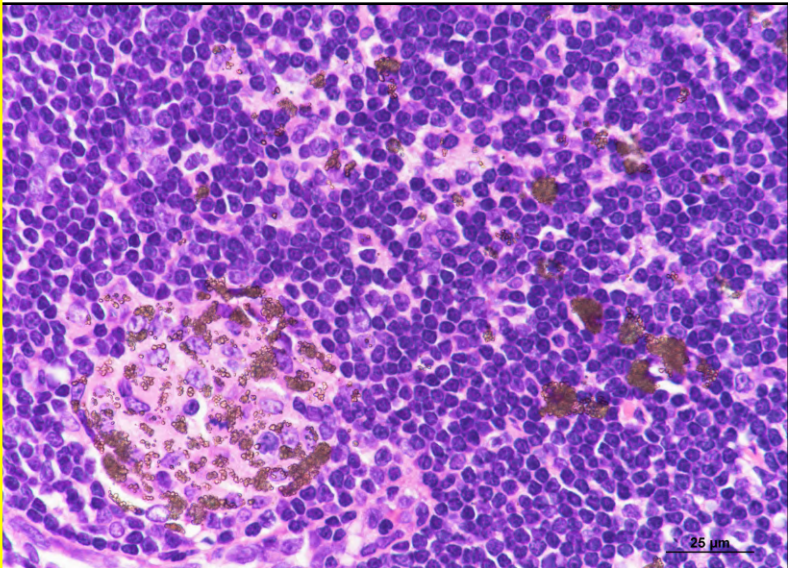

Supplement: Supplementary file 6 — Additional file 6: Fig. S6. Representative microscopic photographs of mediastinal lymph nodes. A typical loupe image of the entire mediastinal lymph node and magnified images of each lymph node from a female control rat (A) and a female rat exposed to 50 mg/m3 (B) are shown. [file 12989_2022_498_MOESM6_ESM.pdf]

Fig. S7

Bronchial lineage markers (50 mg/m<sup>3</sup>)

A

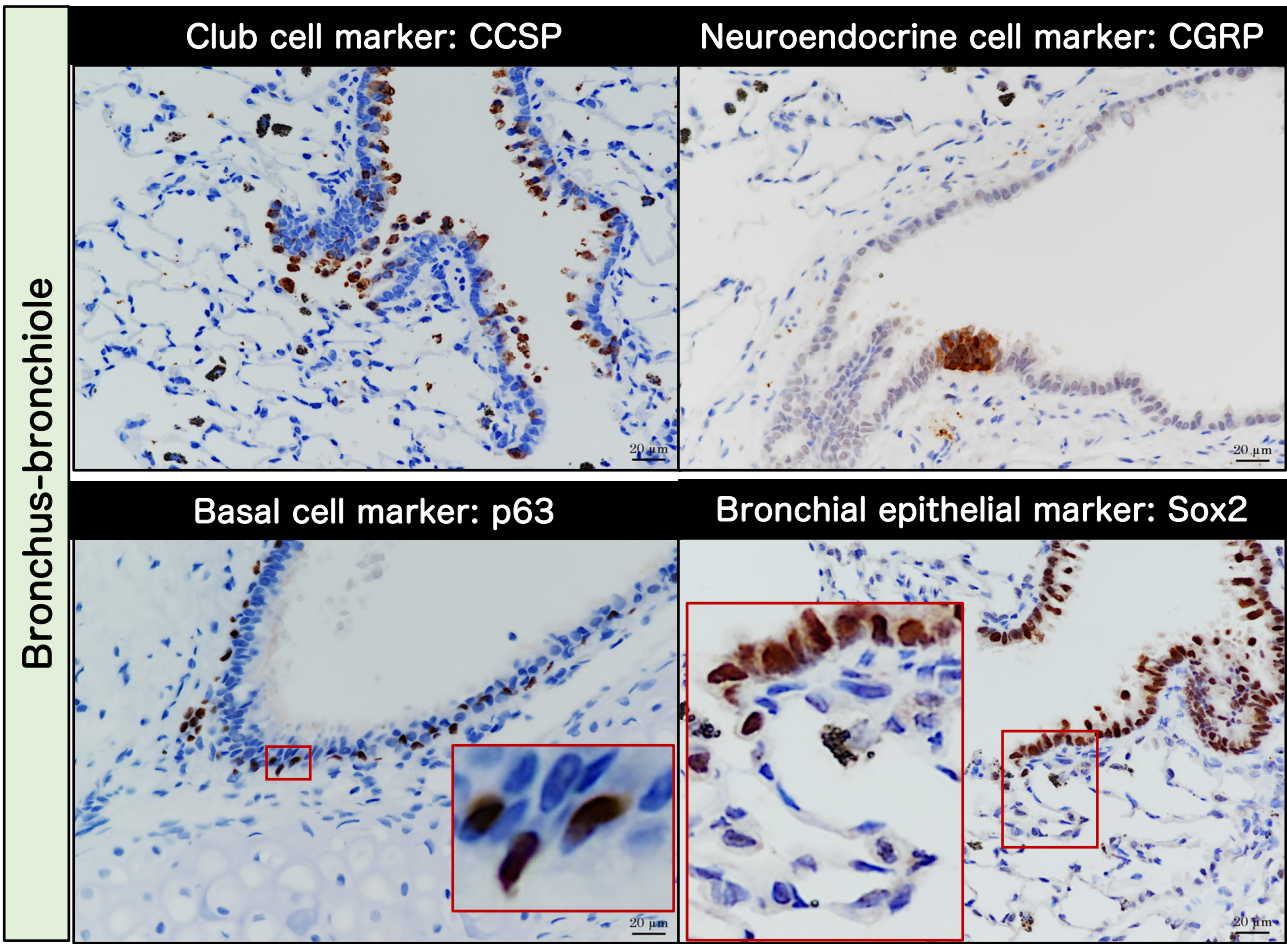

B

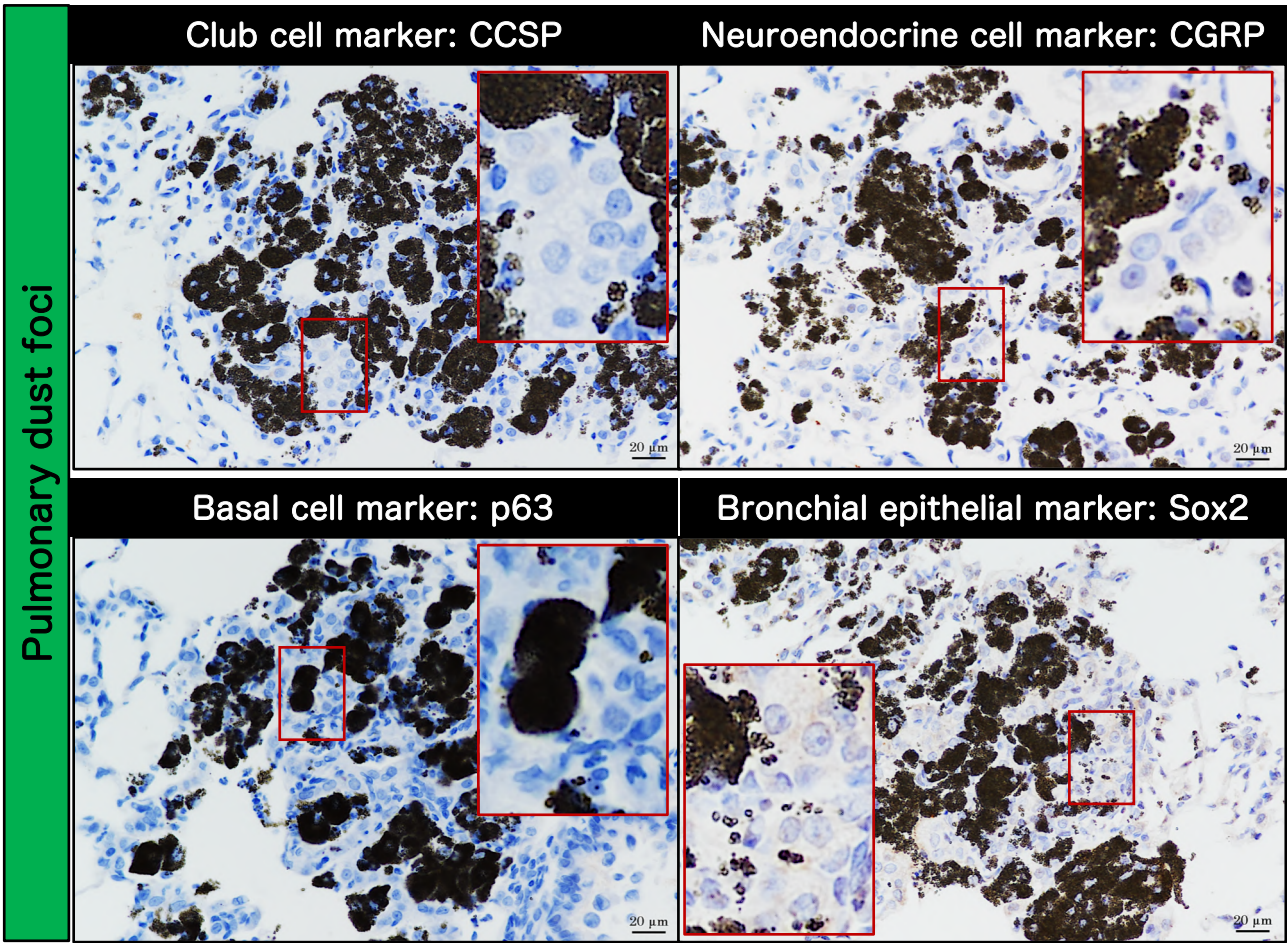

Supplement: Supplementary file 7 — Additional file 7: Fig. S7. Immunohistochemistrical characteristics of bronchial lineage markers in tissue surrounding a lesion and in pulmonary dust foci. Representative immunohistochemical staining images of the club cell marker club cell secretory protein (CCSP), neuroendocrine cell marker calcitonin gene-related peptide (CGRP), basal cell marker p63, and bronchial epithelial lineage marker SRY-Box Transcription Factor 2 (Sox2) in the bronchus-bronchiole (A) and in pulmonary dust foci (B). [file 12989_2022_498_MOESM7_ESM.pdf]

Fig. S8

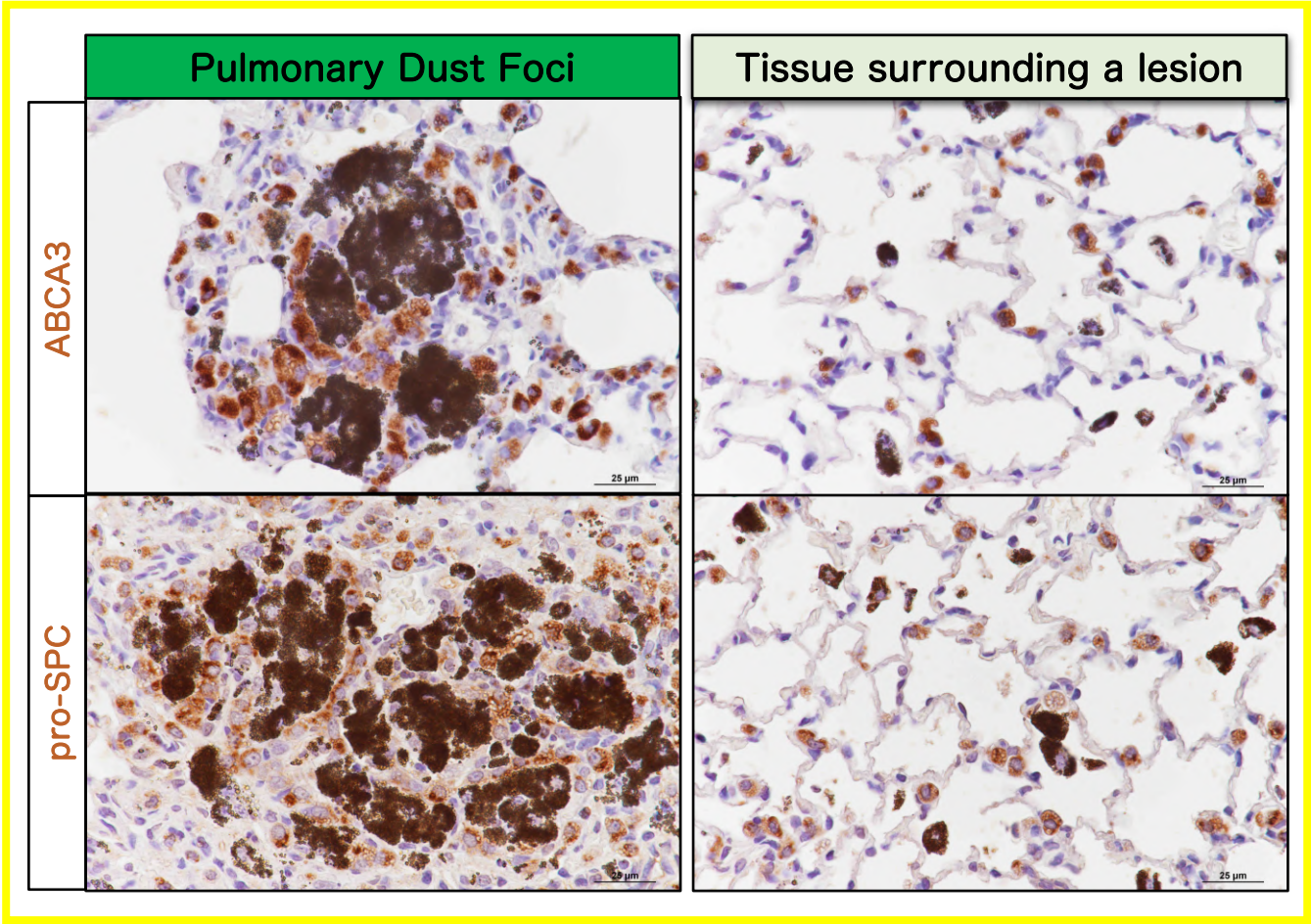

Supplement: Supplementary file 8 — Additional file 8: Fig. S8. Additional AEC2 marker expression in pulmonary dust foci (PDF) and in tissue surrounding a lesion in a rat lung after inhalation exposure to TiO2 NP (50 mg/m3). Representative images of alveolar epithelial type 2 cell (AEC2) markers ABCA3 and proSPC in PDF and tissue surrounding a lesion. [file 12989_2022_498_MOESM8_ESM.pdf]

Fig. S9

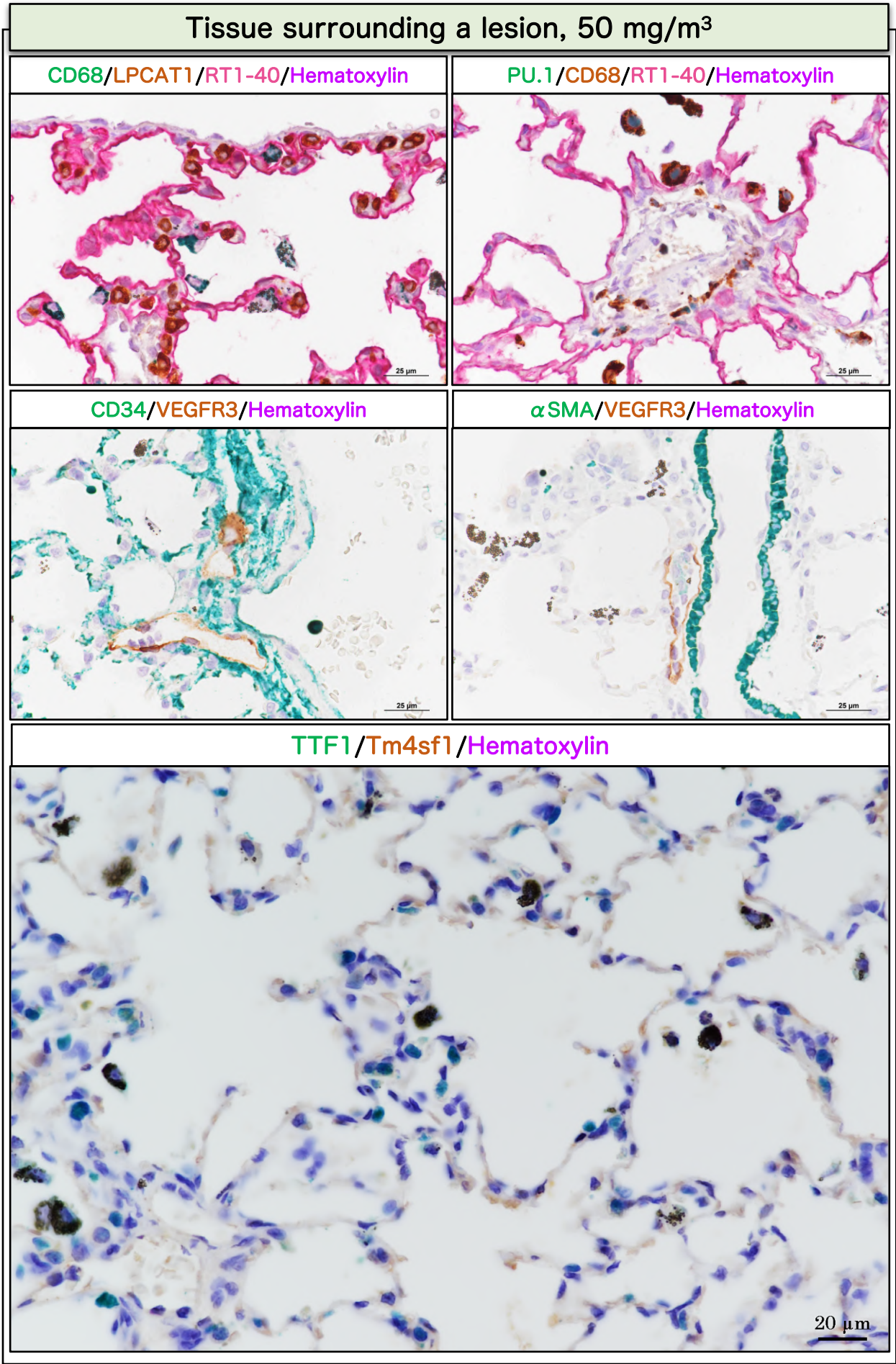

Supplement: Supplementary file 9 — Additional file 9: Fig. S9. Immunohistochemical characteristics in tissue surrounding a lesion in rat lungs after inhalation exposure to TiO2 NP (50 mg/m3): same rat as in figure 9. Representative images of staining sets similar to figure 9 in tissue surrounding a lesion (normal tissue). [file 12989_2022_498_MOESM9_ESM.pdf]

Fig. S10

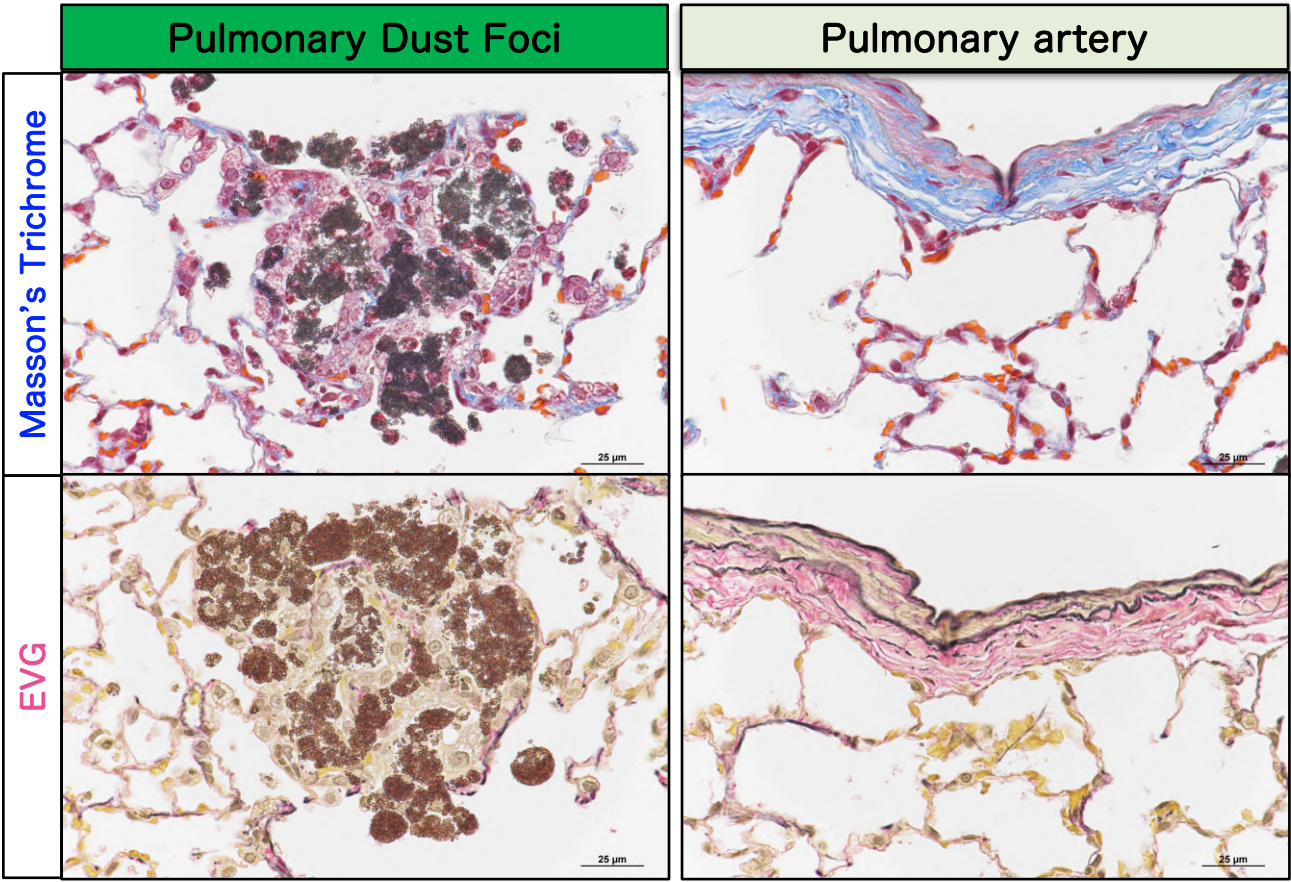

Supplement: Supplementary file 10 — Additional file 10: Fig. S10. Representative microscopic photographs of Masson’s trichrome and EVG staining in pulmonary dust foci and a pulmonary artery. Both stains were strongly positive in the arterial wall within the lung (right), but negative in the interstitium of the pulmonary dust foci (left). Abbreviations: EVG, Elastica Van Gieson. [file 12989_2022_498_MOESM10_ESM.pdf]

Fig. S11

A

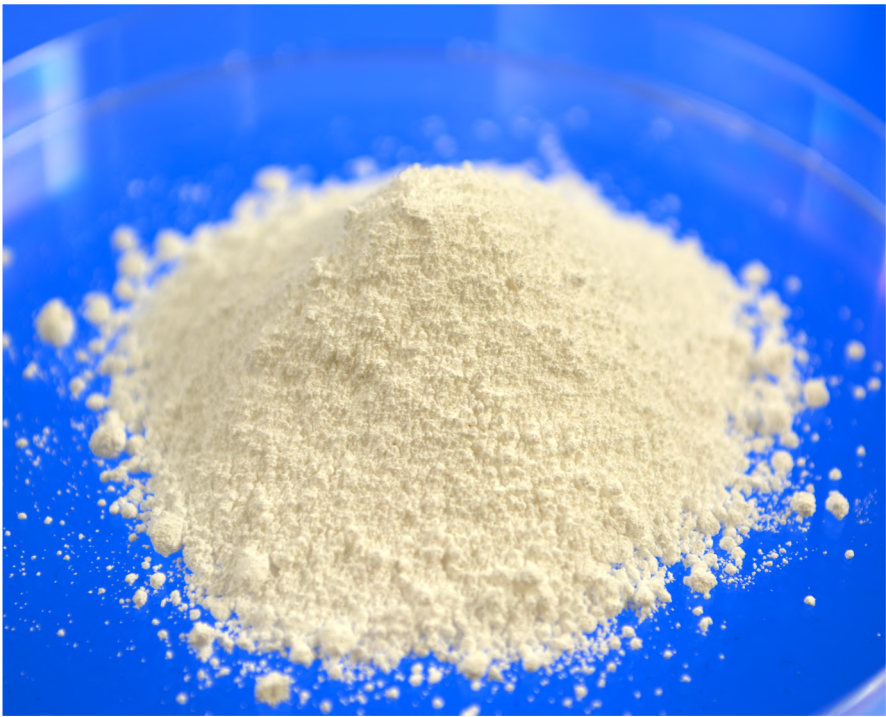

B

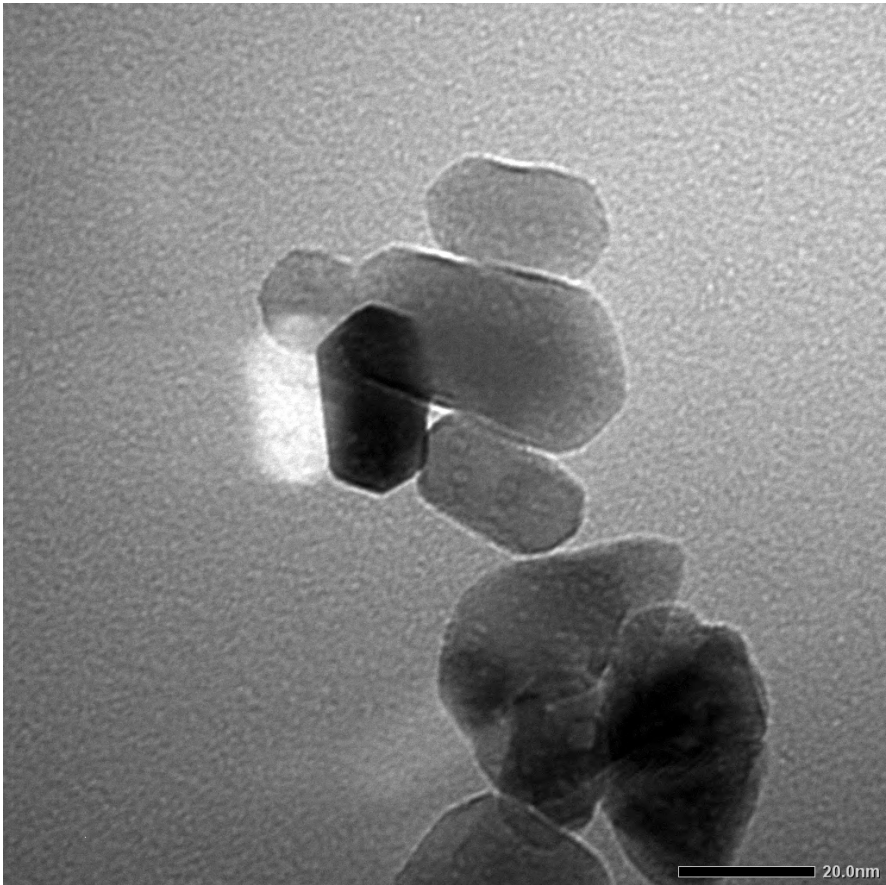

Supplement: Supplementary file 11 — Additional file 11: Fig. S11. Representative macroscopic and TEM images of TiO2 NP. A: Macroscopic image. B; TEM image. [file 12989_2022_498_MOESM11_ESM.pdf]
