# Supplementary material for: Pulmonary dust foci as rat pneumoconiosis lesion induced by titanium dioxide nanoparticles in 13-week inhalation study
Source: Part Fibre Toxicol. 2022 Sep 14;19:58. doi: 10.1186/s12989-022-00498-3 (PMC9472424; doi:10.1186/s12989-022-00498-3)
Supplement: Supplementary file 12 — Additional file 12: Fig. S12. Design of the animal experimental protocol used in this study. [file 12989_2022_498_MOESM12_ESM.pdf]

Fig. S12

Experimental protocol
(inhalation study: dose-response)

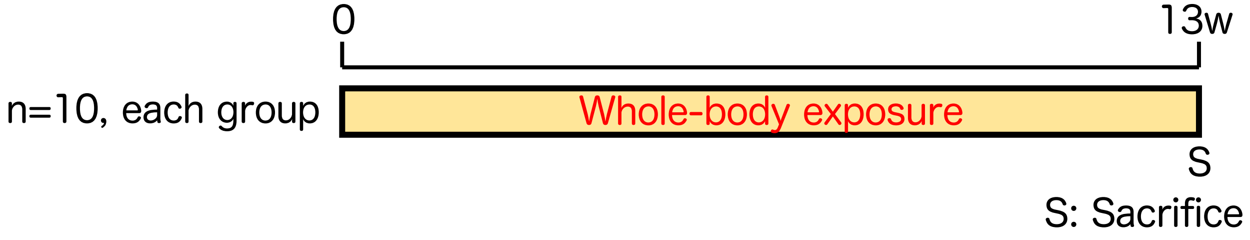

Animal: F344/DuCrlCrlj rat, 6-week-old
male (n=10 each group), female (n=10 each group), total n=100
Test compound: Anatase type titanium dioxide nanoparticles (primary particle size: 30 nm)
Exp. Conc.: 6hr/day, 5 day/week, 0, 6.3, 12.5 , 25 and 50 mg/m^3
